# Short oligogalacturonides induce pathogen resistance-associated gene expression in *Arabidopsis thaliana*

**DOI:** 10.1186/s12870-016-0959-1

**Published:** 2017-01-19

**Authors:** Pär Davidsson, Martin Broberg, Tarja Kariola, Nina Sipari, Minna Pirhonen, E. Tapio Palva

**Affiliations:** 1 Department of Biosciences, Division of Genetics, University of Helsinki, Helsinki, Finland; 2 Department of Agricultural Sciences, University of Helsinki, Helsinki, Finland; 3 School of Biological Sciences, Bangor University, Bangor, Wales, UK

**Keywords:** Plant signaling, *Arabidopsis thaliana*, Oligogalacturonides, OG, Trimers, Transcriptomics, Defense induction, Growth inhibition, Disease resistance, *Pectobacterium carotovorum*, *Botrytis cinerea*

## Abstract

**Background:**

Oligogalacturonides (OGs) are important components of damage-associated molecular pattern (DAMP) signaling and influence growth regulation in plants. Recent studies have focused on the impact of long OGs (degree of polymerization (DP) from 10–15), demonstrating the induction of plant defense signaling resulting in enhanced defenses to necrotrophic pathogens. To clarify the role of trimers (trimeric OGs, DP3) in DAMP signaling and their impact on plant growth regulation, we performed a transcriptomic analysis through the RNA sequencing of *Arabidopsis thaliana* exposed to trimers.

**Results:**

The transcriptomic data from trimer-treated Arabidopsis seedlings indicate a clear activation of genes involved in defense signaling, phytohormone signaling and a down-regulation of genes involved in processes related to growth regulation and development. This is further accompanied with improved defenses against necrotrophic pathogens triggered by the trimer treatment, indicating that short OGs have a clear impact on plant responses, similar to those described for long OGs.

**Conclusions:**

Our results demonstrate that trimers are indeed active elicitors of plant defenses. This is clearly indicated by the up-regulation of genes associated with plant defense signaling, accompanied with improved defenses against necrotrophic pathogens. Moreover, trimers simultaneously trigger a clear down-regulation of genes and gene sets associated with growth and development, leading to stunted seedling growth in Arabidopsis.

**Electronic supplementary material:**

The online version of this article (doi:10.1186/s12870-016-0959-1) contains supplementary material, which is available to authorized users.

## Background

Necrotrophic phytopathogens, which are ubiquitous in nature, are represented among bacteria and fungi. Many agriculturally important diseases, such as soft rot and blackleg, are caused by bacterial necrotrophs [1, 2], while necrotrophic fungi are causative agents of devastating gray molds and rusts, among others [3, 4]. Enterobacterial soft-rot pathogens of the genus *Pectobacterium* include broad host-range pathogens that cause disease in a variety of plant species and economically important crops, such as potato [1, 5, 6]. Similar to many other necrotrophic brute force pathogens, resistance to broad host-range Pectobacteria is complex and does not appear to involve single resistance genes [7–9]. Instead, general plant innate immunity systems, including salicylic acid (SA)- and jasmonic acid/ethylene (JA/ET)-mediated defenses, are triggered by conserved pathogen-associated molecular patterns (PAMPs) [10–15]. In addition to bacterial necrotrophs, also fungi cause severe pre- and post-harvest losses to crops worldwide [16]. Of these, the wide broad host-range gray mold pathogen *Botrytis cinerea,* capable of infecting more than 200 plant species, is one of the most comprehensively studied necrotrophic fungus. Similar to Pectobacteria, *B. cinerea* uses enzymes to break down cell walls to access the host tissue. In Arabidopsis, enhanced plant resistance to *Botrytis* seems to be independent of the phytohormones SA and JA, but rather dependent on ET, PAD3 and the accumulation of the phytoalexin camalexin [17, 18].

In addition to PAMPs, damage-associated molecular patterns (DAMPs) play a vital role in defense activation against bacterial and fungal necrotrophs (i.e. Pectobacteria and Botrytis). Plants perceive DAMPs, such as plant cell wall fragments released by the action of plant cell wall-degrading enzymes (PCWDEs) secreted by these pathogens, as signals of damage or modified self [10, 19]. PCWDEs are among the central virulence determinants employed by necrotrophic phytopathogens for the maceration of host tissue and the release of nutrients [20]. Pectin is a major component of the primary plant cell wall matrix. It is a complex heteropolysaccharide composed of galacturonan residues and a prominent target of PCWDEs [21, 22]. Both Pectobacteria and Botrytis employ PCWDEs for the degradation of pectin, central of which are; pectin methylesterases (Pem) and pectin lyases (Pnl), which directly operate on pectin polymers and polygalacturonases (Peh) and pectate lyases (Pel), which operate on pectate (de-esterified pectin) [20–24].

Major end products of the degradation of pectin by PCWDEs are oligogalacturonides (OGs) with varying degrees of polymerization (DP). These OG fragments act as potent DAMPs, capable of triggering plant defense signaling [25–29]. In addition to plant responses to pathogens, OGs regulate plant growth and development [30]. Consequently, OGs play an important biological role as signaling molecules, but the complexity of both OG fragments and the responses generated has resulted in OGs being difficult to study [29, 31]. Early studies on plant response to OGs were often limited to specific molecular targets or processes, including; the production of plant hormones, such as ET and JA, or the expression of specific defense-related genes [26, 28, 32–34]. More recent transcriptomics and functional genetic analyses have enabled systems-level studies of plant responses to OGs and the characterization of the OG-responsive transcriptome in Arabidopsis [17, 30, 35]. These studies have suggested that long OGs (DP > 10) are the most effective in modulating signaling involved in plant defense responses, with short OGs having little or no effect [17, 30, 35, 36]. In part, recent studies have focused on long OGs because the only identified receptor, WAK1, is reported to bind to and be stimulated by long OGs [37, 38].

However, previous studies by us and others have suggested that also short OGs (DP < 10) impact plant defense [28, 39] and development [40]. For example, short OGs of DP2-5 induced a strong expression of a gene involved in JA biosynthesis in Arabidopsis [28]. Furthermore, short OGs (DP4-6, DP2 and DP1-7, respectively) induced genes involved in pathogen response and defense in potato and tomato and the synthesis of phytohormones in tobacco and tomato [28, 39, 41–43]. Moloshok et al. [44] demonstrated the induction of a proteinase inhibitor in response to dimeric OGs in tomato seedlings.

To clarify the impact of short OGs on plant signaling associated with defense and development, we performed an RNA sequencing assay using trimers (trimeric OGs, DP3) as models for short OGs. Trimers were chosen as model as during the natural growth phases of Arabidopsis trimers are present during senescence as well as after cell wall digestion by pectinases [45]. Furthermore, in plant pathogen interactions it has been demonstrated that trimers are produced in tomato fruit after infection with B. cinerea [46]. For bacterial pathogens it has been observed that the pectolytic enzymes from various species generate trimers from pectin [47–51], and we have previously shown that commercially available trimers have a similar effect when applied exogenously to plant tissue, as does culture filtrate from P. carotovorum and polygalacturonic acid degraded with pectolytic enzymes.

We further compared the sequencing results to those of previous studies detailing the global transcriptomic effects of long OGs (DP > 10) on Arabidopsis [17, 30, 35]. The transcriptomic data obtained by RNA sequencing suggests that trimers clearly induce the expression of a number of genes involved in plant defense, while genes involved in growth, biosynthetic pathways and development are down-regulated. The up-regulation of defense-associated and OG-responsive genes identified in this study was comparable to previous studies in which in vitro*-*grown Arabidopsis seedlings have been treated with long OGs [17, 35]. However, the modulation of gene expression induced by trimers was generally not as strong as that induced by long OGs. To confirm the transcriptomic data, we performed quantitative RT-PCR (qRT-PCR) analyses of genes previously associated with OG signaling [17, 35]. In addition, we conducted phenotypic analyses for the trimer-induced enhancement of pathogen resistance and the inhibition of plant growth. Importantly, pre-treatment with trimers or long OGs induced enhanced resistance to the necrotrophic pathogens *Pectobacterium carotovorum* at a similar degree, and retarded seedling growth in Arabidopsis. Further, both long OGs and trimers were able to induce phosphorylation of MPK3 and MPK6, indicating activation of signaling pathways involved in recognition of elicitors. These results suggest that trimers are potent elicitors of plant defenses, putatively leading to the re-allocation of plant resources from growth and development towards the induction of defense.

## Results

### Short OGs have a significant impact on the arabidopsis transcriptome

Previous studies have characterized the responses triggered in Arabidopsis to exogenously applied oligogalacturonides with DP > 10 at the transcriptome level during the early stages of signaling [30, 35]. In contrast, with the exception of the expression of a few specific genes, the potential of trimers to trigger plant defense signaling has not been characterized at the transcriptome level. In some studies, trimers have failed to elicit specific defense gene expression, whereas in other studies, the up-regulation of specific defense-related genes has been observed [28, 52]. To address this apparent controversy, we characterized the Arabidopsis transcriptome by RNA sequencing in response to trimers. The RNA sequencing data obtained in this study revealed significant differences in the gene expression of plants treated with trimers (183 genes down-regulated and 517 genes up-regulated) compared to plants subjected to mock treatment (Additional file 1: Table S2).

### Comparative meta-data analysis of transcriptomes in response to trimers and long OGs

**Fig. 1:**
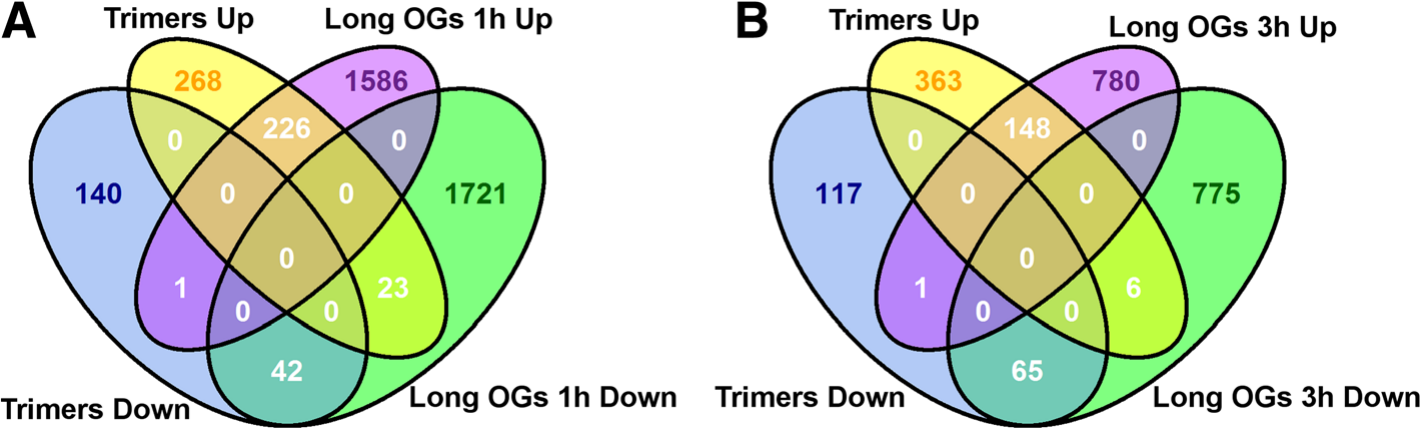
Global gene expression data from trimer-treated seedlings exhibit similarities and differences with those from long-OG-treated seedlings at different time points. Venn diagram comparison of the up- and down-regulated genes based on the RNA sequencing data between trimer (trimers in the figure) pretreatment and treatment with long OGs at 1 h **a** and long OGs at 3 h **b** compared to mock treatment

To elucidate differences and overlaps in plant responses to OGs with different DPs, we compared the transcriptome data obtained by RNA sequencing from trimer-treated samples with the corresponding published transcriptome data from microarray analyses of long OG (DP > 10)-treated samples [17, 30, 35]. We combined RNA-sequencing data from the 3 h trimer treatment with transcriptomic studies of the 1 h and 3 h treatments using long OGs and in vitro Arabidopsis seedlings as described by Denoux et al. [35] and Ferrari et al. [17]. Intriguingly, there was a significant degree of overlap between the studies (Fig. 1).

**Fig. 2:**
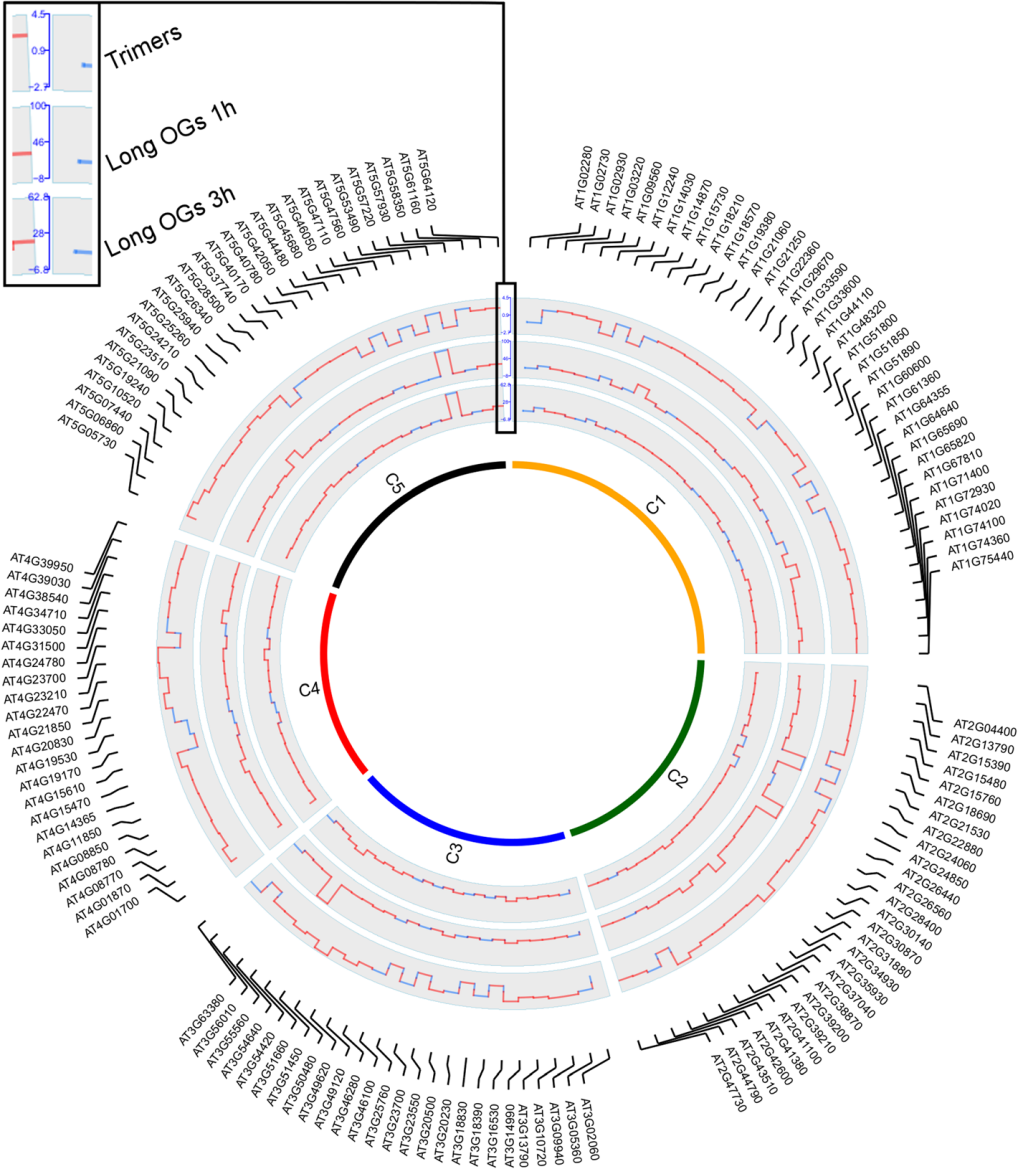
Trimers and long OGs alter the expression of common genes. Circular map of the expression fold changes of 140 genes significantly altered in expression following treatment with trimers and long OGs across two different studies. The fold change map is in the form of a histogram. C1–5 in the figure indicate chromosomes 1–5 of *A. thaliana*

A subsequent comparative analysis of data from all three treatments (trimers at 3 h and long OGs at 1 h and 3 h) identified 140 genes that exhibited significant expression changes in response to treatment with long OGs and trimers (Additional file 2: Table S3, Fig. 2). Of the 140 genes with altered expression across all of the OG treatments, 24 genes were down-regulated and 108 genes were up-regulated. A total of 8 genes differed in up/down-regulation in at least one experiment. We further performed hierarchical clustering (complete agglomeration) based on the expression values of genes triggered by both long and short OGs. Gene expression triggered by the 3 h treatment with trimers and long OGs at 3 h was the most similar across all 3 treatments. These data provide further support for a considerable overlap in the effect of the trimers and long OGs on gene expression.

Moscatiello et al. [30] studied the effects of long OGs (DP > 10) on Arabidopsis in cell culture. When the transcriptomic data from this study were included in our comparative analysis, only the expression of ten genes was significantly altered across all four experiments (Table 1). The ten genes with altered expression included defense-associated genes, such as peroxidases. Although all of the ten common OG-associated genes were down-regulated in the study by Moscatiello et al. [30], they were up-regulated in the other studies.

**Table 1:** Genes with altered expression across different studies that examined the response to trimers and long OGs

Id	Description	Trimers	Long OGs 1 h	Long OGs 3 h	Long OGs 2 h
AT2G30870	Glutathione S-transferase PHI 10/ERD13	1.43	2.11	2.19	−2.32
AT1G33590	Leucine-rich repeat (LRR) family protein	1.43	3.70	2.20	−5.01
AT4G08770	Peroxidase superfamily protein	1.50	3.56	4.59	−5.25
AT4G38540	FAD/NAD(P)-binding oxidoreductase family protein	1.54	2.10	1.94	−2.91
AT3G49120	Peroxidase CB	1.60	2.27	1.84	−4.66
AT5G40170	Receptor like protein 54	1.61	3.91	1.37	−2.21
AT3G05360	Receptor like protein 30	1.70	8.04	6.51	−2.15
AT4G08780	Peroxidase superfamily protein	1.77	1.92	2.48	−5.96
AT3G13790	Glycosyl hydrolases family 32 protein	1.81	3.70	4.88	−6.31
AT2G34930	Disease resistance family protein/LRR family protein	4.09	5.95	3.59	−4.57

Genes significantly affected by trimers and long OGs across three different *A. thaliana* transcriptome studies are presented: this study, long OGs at 1 h and 3 h and long OGs at 2 h in a cell-culture [17, 30, 35]. Numeric values indicate the fold change compared to a mock treatment

In conclusion, a comparative gene expression analysis of studies using short and long OGs suggests that several defense-associated genes are up-regulated by both types of OGs in similar experiments. Furthermore, the expression of genes altered both in this study and those published by Ferrari et al. [17] and Denoux et al. [35] at the 3 h time point was generally similar to data from the 1 h time point. In addition to time point-dependent changes, the impact of OGs depends on the type of experiment, as suggested by the gene expression analysis by Moscatiello et al. [30], which was performed using Arabidopsis cells in a suspension culture. In our study, the results from the global transcriptomic analysis resemble the previously characterized effects of long OGs, suggesting that trimers have a significant impact on the expression of hundreds of plant genes, many similar to those affected by long OGs.

### Gene sets associated with pathogen response and hormone signaling are up-regulated while those associated with metabolic and developmental pathways are down-regulated across studies of OG treatment

**Fig. 3:**
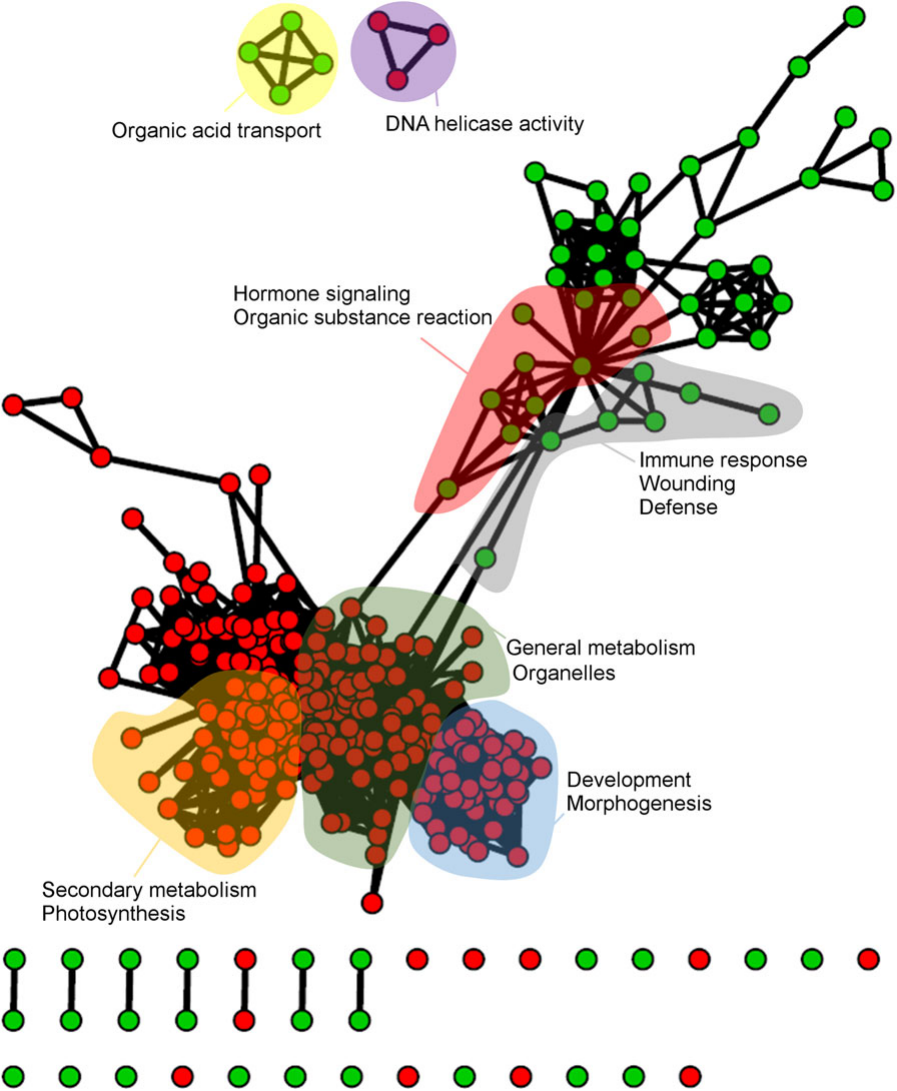
Trimers cause an up-regulation of the immune response and hormone signaling together with the down-regulation of general metabolism and development. The GSEA map was made using the cytoscape plugin Enrichment map (Bader lab), depicting the results of treatment with trimers using FDR < 0.01 as the cut-off [92]. *Colored* areas signify gene set clusters of with common functional themes. *Green* indicates up-regulated gene sets, and *red* indicates down-regulated gene sets

A gene set enrichment analysis (GSEA) was performed using transcriptome data from our RNA sequencing and from previous microarray studies characterizing plant response to long OGs (DP > 10). Gene sets were obtained from the Plant Gene Set Enrichment Analysis Toolkit (http://structuralbiology.cau.edu.cn/PlantGSEA/database/Ara.DetailInfo), where references for all of the literature- and database-derived gene sets identified in the following GSEA can be obtained [53]. From the GSEA output of the trimer/mock RNA sequencing data, we identified 247 down-regulated gene sets and 73 up-regulated gene sets using an FDR of <0.01 as the cut-off (Additional file 3: Table S4, Fig. 3).

This data analysis suggests a trend of trimer-mediated up-regulation of biotic defense-related gene sets. These include gene sets induced in Arabidopsis after inoculation with *Xanthomonas campestris* pv. *campestris* and gene sets induced by *Myzus persicae* or *Plutella xylostella* [54–56]. Based on the gene ontology (GO) database (http://geneontology.org/, the Gene Ontology Consortium), the up-regulated gene sets included jasmonic acid biosynthesis (GO:0009695), ethylene response (GO:0009723), oxylipin biosynthesis (GO:0031408), response to wounding (GO:0009611) and immune effector processes (GO:0002252). The down-regulated gene sets included cellular component biogenesis (e.g., GO:004225 - ribosome biogenesis and GO:0070271 - protein complex biogenesis), organelle organization (e.g., GO:0009657 - plastid organization, GO:0006996 - organelle organization, GO:0009668 - plastid membrane organization and GO:0009658 - chloroplast organization), energy metabolism (e.g., GO:0015979 - photosynthesis, GO:0005996 - monosaccharide metabolic process and GO:0019684 - photosynthesis light reaction) and development (e.g., GO:0009790 - embryo development, GO:0048316 - seed development and GO:0044085 - cellular component biogenesis).

**Fig. 4:**
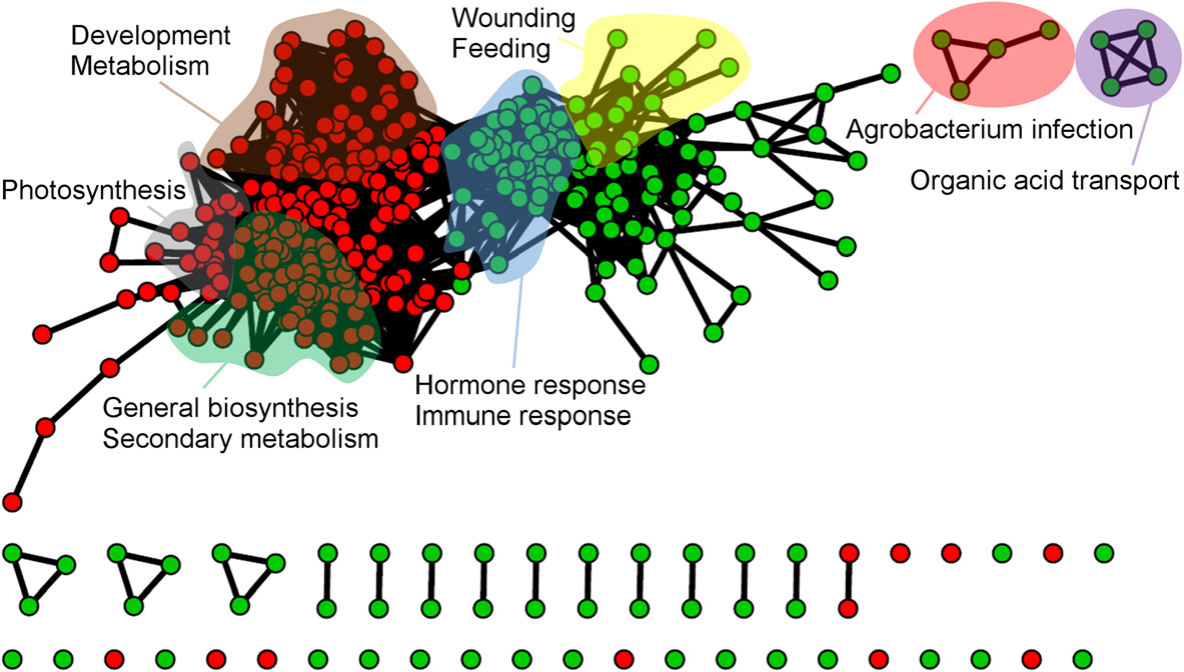
Both trimers and long OGs trigger the up-regulation of immune and stress responses and the down-regulation of metabolism. The GSEA map was made using the cytoscape plugin Enrichment map (Bader lab), depicting the results of combined data for trimers and long OGs (combined gene expression data for trimers and long OGs at 3 h as reported by Denoux et al. 2008) using a *P*-value cut-off of <0.05. *Colored* areas indicate gene set clusters of with common functionality. *Green* indicates up-regulated gene sets, and *red* indicates down-regulated gene sets

A GSEA analysis was performed on genes that were similarly regulated by trimers and long OGs at 3 h and using only gene sets from the GO database with a GSEA *P*-value cut-off of <0.05. A total of 213 gene sets were found to be up-regulated, and 244 were down-regulated (Additional file 4: Table S5). By further filtering the GSEA output by only including expression data of genes similarly triggered by long OGs at 1 h, we found 94 gene sets to be up-regulated and 92 to be down-regulated. In the filtered GSEA output, we detected a trend for the up-regulation of defense- (e.g., GO:0009620 - response to fungus, GO:0009626 - plant hypersensitive response and GO:0045087 - innate immune response) and JA-related gene sets (GO:0009694 - jasmonic acid metabolism and GO:0009695 - jasmonic acid biosynthesis). In contrast, gene sets involved in photosynthesis (GO:0015979) and in the biosynthesis of isoprenoids (GO:0008299), phospholipids (GO:0008654) and pigments (GO:0046148) were down-regulated (Tables 2 and 3, Additional file 5: Table S6, Fig. 4).

**Table 2:** Trimers and long OGs induce gene sets associated with phytohormones, stress and defense signaling

Gene set	ID	Size	NES	*P*-value
RESPONSE_TO_ORGANIC_SUBSTANCE	GO:0010033	59	2.11	0.00
OXYLIPIN_METABOLIC_PROCESS	GO:0031407	9	2.10	0.00
OXYLIPIN_BIOSYNTHETIC_PROCESS	GO:0031408	9	2.05	0.00
JASMONIC_ACID_METABOLIC_PROCESS	GO:0009694	8	1.92	0.00
JASMONIC_ACID_BIOSYNTHETIC_PROCESS	GO:0009695	8	1.90	0.00
RESPONSE_TO_CARBOHYDRATE_STIMULUS	GO:0009743	27	1.84	0.00
CELLULAR_RESPONSE_TO_CHEMICAL_STIMULUS	GO:0070887	33	1.78	0.02
RESPONSE_TO_CHITIN	GO:0010200	26	1.78	0.02
FATTY_ACID_BIOSYNTHETIC_PROCESS	GO:0006633	12	1.75	0.02
CELLULAR_RESPONSE_TO_STIMULUS	GO:0051716	49	1.74	0.02
IMMUNE_SYSTEM_PROCESS	GO:0002376	49	1.70	0.03
CELLULAR_RESPONSE_TO_STRESS	GO:0033554	45	1.67	0.03
RESPONSE_TO_ETHYLENE_STIMULUS	GO:0009723	14	1.66	0.03
IMMUNE_RESPONSE	GO:0006955	49	1.64	0.03
RESPONSE_TO_FUNGUS	GO:0009620	32	1.64	0.03
INNATE_IMMUNE_RESPONSE	GO:0045087	49	1.63	0.02
RESPONSE_TO_CYCLOPENTENONE	GO:0010583	11	1.61	0.04
PROGRAMMED_CELL_DEATH	GO:0012501	26	1.61	0.04
PLANT-TYPE_HYPERSENSITIVE_RESPONSE	GO:0009626	26	1.61	0.03
HOST_PROGRAMMED_CELL_DEATH_INDUCED_BY_SYMBIONT	GO:0034050	26	1.60	0.04

The 20 most significantly up-regulated gene sets based on the GO database are presented, as determined by a GSEA on the GenePattern platform using the combined trimer and long OG gene expression data

**Table 3:** Trimers and long OGs down-regulate gene sets associated with organelle organization, photosynthesis and metabolic processes

Gene set	Identity	Size	NES	*P*-value
RNA_METABOLIC_PROCESS	GO:0016070	13	-2.78	0
CHLOROPLAST_PART	GO:0044434	17	-2.69	0
PIGMENT_BIOSYNTHETIC_PROCESS	GO:0046148	6	-2.66	0
NCRNA_METABOLIC_PROCESS	GO:0034660	6	-2.66	0
PYRUVATE_METABOLIC_PROCESS	GO:0006090	11	-2.66	0
ISOPRENOID_METABOLIC_PROCESS	GO:0006720	11	-2.66	0
ISOPENTENYL_DIPHOSPHATE_BIOSYNTHETIC_PROCESS	GO:0009240	11	-2.65	0
CELLULAR_CARBOHYDRATE_METABOLIC_PROCESS	GO:0044262	22	-2.65	0
ISOPENTENYL_DIPHOSPHATE_BIOSYNTHETIC_PROCESS, MEVALONATE-INDEPENDENT_PATHWAY	GO:0019288	11	-2.64	0
PIGMENT_METABOLIC_PROCESS	GO:0042440	6	-2.64	0
PLASTID_PART	GO:0044435	17	-2.64	0
CELLULAR_GLUCAN_METABOLIC_PROCESS	GO:0006073	6	-2.64	0
PLASTID	GO:0009536	28	-2.63	0
ISOPENTENYL_DIPHOSPHATE_METABOLIC_PROCESS	GO:0046490	11	-2.61	0
CELLULAR_POLYSACCHARIDE_METABOLIC_PROCESS	GO:0044264	6	-2.60	0
GLUCAN_BIOSYNTHETIC_PROCESS	GO:0009250	6	-2.59	0
POLYSACCHARIDE_METABOLIC_PROCESS	GO:0005976	6	-2.59	0
GLYCERALDEHYDE-3-PHOSPHATE_METABOLIC_PROCESS	GO:0019682	11	-2.58	0
CHLOROPLAST	GO:0009507	27	-2.58	0

The 20 most significantly down-regulated gene sets based on the GO database are presented, as determined by a GSEA on the GenePattern platform using the combined trimer and long OG gene expression data

To examine differences in the responses to trimers or long OGs, the GSEA (FDR < 0.01) results for long OGs at 3 h (using a *P*-value of <0.01 and fold changes of > log_2_ 0.5 and < log_2_ 0.5) and trimers were compared by selecting gene sets that were affected by one but not the other (Additional file 6: Table S7). Trimer-specific gene sets that were not found to be significantly affected by the long OGs at 3 h were involved in, for example, developmental processes (GO:0048869-cellular developmental process, GO:0048364-root development and GO:0051301-cell division). The gene sets that were up-regulated by long OGs but not by trimers (Additional file 7: Table S8) were involved in respiratory burst (GO:0045730) and response to microbes (GO:0009617 – response to bacterium, GO:0009627 – systemic acquired resistance and GO:0009814 – defense response incompatible reaction).

A pathway analysis was performed for the additional characterization of how trimers and long OGs affect genes involved in the plant-pathogen response and hormone signaling. Both trimers and long OGs induce the expression of genes triggered by fungal and bacterial pathogens. According to the plant-pathogen pathway map of the KEGG database, these genes include *BAK1* (encoding a leucine-rich receptor-like kinase with a central role in the PAMP-response triggered by flagellin), calmodulin-like (*CML)* genes and genes encoding calcium-dependent protein kinases (*CDPK*s; Additional file 8: Figure S1). Characterization of the hormonal signaling pathways induced by both 3 h treatments (trimers and long OGs) revealed the induction of genes associated with ET and JA responses and the SA response.

The results of the GSEA and pathway analyses of the effect of short and long OG treatments suggest a trade-off in the priorities for plant resource allocation. The expression of genes associated with the defense-related JA, ET and SA signaling pathways was enhanced, while the expression of genes involved in the gibberellic acid and cytokinin pathways, associated with development and growth, were mainly down-regulated (Additional file 9: Figure S2). Thus, the global transcriptomic data suggest that trimers induce a significant response from gene sets and pathways associated with pathogen defense, phytohormone production and plant development.

### The expression of OG-responsive marker genes correlates with the short-OG-triggered induction of defense-related pathways

**Fig. 5:**
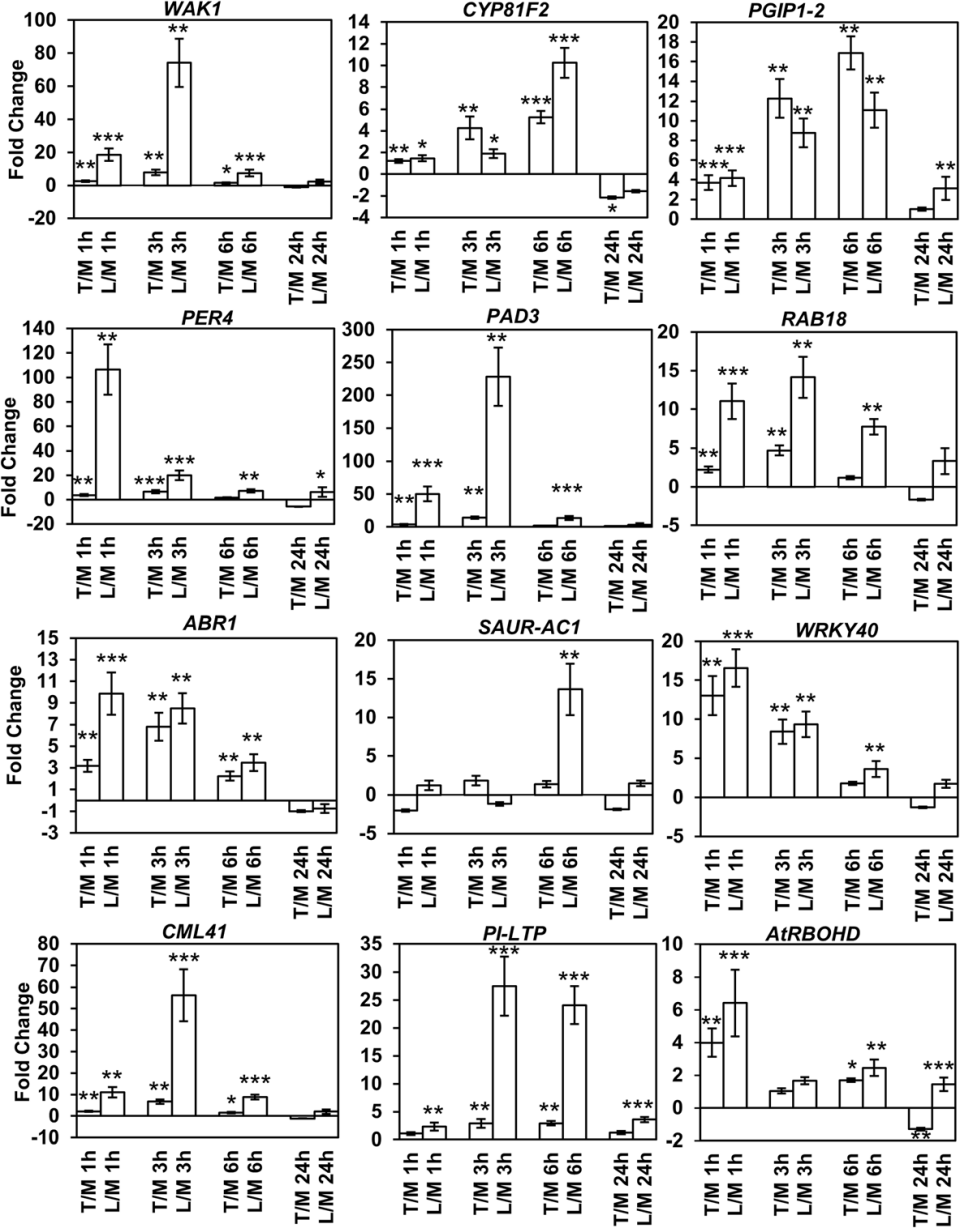
Trimers trigger the up- and down-regulation of genes involved in defense, phytohormone signaling and the OG response, similarly to the long-OG mix (DP > 8). The results from the qRT-PCR gene expression analysis are presented. T/M indicates trimer-treated plants compared to mock treated plants, and L/M indicates plants treated with the long-OG mix (DP > 8) compared to those treated with the mock suspension. A total of 4 time points (1, 3, 6 and 24 h) were analyzed for each gene. Statistical analysis using Student’s *t*-test is indicated by *asterisks* in the figure (1 asterisk = *P* < 0.05, 2 asterisks = *P* < 0.01, and 3 asterisks = *P* < 0.001)

To confirm the trimer-triggered transcriptomic changes indicated by RNA sequencing and to further compare the impact of trimers on the specific gene-expression changes induced by long OGs (DP > 10) in Arabidopsis, we performed qRT-PCR and assayed the expression of selected genes previously shown to be induced by long OGs, as well as those indicating the activation of phytohormone signaling pathways [17, 35]. Plants were mock treated, treated with trimers or treated with a commercial long-OG mix (DP8-19) (Fig. 5).

Results from the qRT-PCR analysis confirmed, in general, activity similar to that observed in the RNA sequencing data. The OG receptor *WAK1* was significantly up-regulated by trimers and to even greater extent by the long-OG mix at 1, 3 and 6 h [35, 37, 38]. The up-regulation of *WAK1* indicates that short OGs are also capable of inducing genes affected by long OGs (Fig. 5).

Since OGs are involved in DAMP signaling in Arabidopsis, we assayed the induction of glucosinolate defenses activated in response to tissue damage [57]. Accordingly, *CYP81F2*, an oxidase involved in glucosinolate modification, was rapidly induced by both trimers and the long-OG mix; trimers triggered stronger expression of this gene at the 3 h time point and the long-OG mix did so at 6 h (Fig. 5).

Furthermore, necrotrophs such as *B. cinerea*, damage plant tissues by secreting various PCWDEs, such as polygalacturonases, thereby releasing OGs from the cell wall [58, 59]. Consequently, *PGIP1–2* (*Polygalacturonase Inhibiting Protein 1–2*) was up-regulated by both trimers and the long-OG mix from 1 to 6 h. Interestingly, compared to long OGs, trimers triggered a clearly stronger induction of this gene at 3 and 6 h. Furthermore, the anionic peroxidase *PER4,* which is required for basal plant resistance to *B. cinerea,* was rapidly induced in response to both trimers and the long-OG mix [60]. However, the expression triggered by the long-OG mix was considerably higher than that induced by trimers, especially at 1 h. In Arabidopsis, the phytoalexin camalexin, originating from the tryptophan pathway, is central for OG-triggered defense to *B. cinerea* [60]. We noted up-regulation of *PAD3 (Phytoalexin Deficient 3)*, which encodes an enzyme required for the biosynthesis of camalexin in response to both trimers and the long-OG mix from 1 to 3 h (Fig. 5). However, the long-OG mix induced clearly higher and longer-lasting expression of this gene compared to induction by trimers.

Damage and the subsequent release of OGs from plant cell walls also modulate phytohormone signaling, such as that mediated by ABA, JA and auxin [61]. We detected clear induction of *RAB18*, a marker for ABA signaling, in response to the long-OG mix and, to a lesser extent, to trimers [62]. On the other hand, *ABR1*, a repressor of ABA responses, was also induced by both trimers and the long-OG mix at 1 and 6 h post-treatment, indicating OG-triggered down-regulation of ABA signaling [63]. Furthermore, *NCED4*, involved in ABA signaling, was down-regulated according to the sequencing data. The rapid induction of the transcription factor *WRKY40* by both types of OGs suggested the same, as well as the simultaneous activation of JA-signaling associated with DAMP response. Here, the trimer treatment displayed a greater effect on the fold change than did the longer OGs. In addition to defense signaling, OGs have also been shown to influence processes involved in plant growth and development [64]. This was also suggested by the RNAseq data, in which the processes related to growth and development were clearly down-regulated. Accordingly, both trimers and the long-OG mix triggered the down-regulation of *SAUR-AC1*, a marker gene for auxin signaling during the early OG response. This, along with the down-regulation of another gene involved in Auxin production, *TAR2*, indicates crosstalk between different hormonal signaling pathways during the early stage of the DAMP response. However, this signaling seems complex in its nature, as *IAA2* was up-regulated.

In response to long OGs, the calmodulin-like gene *CML41* is significantly up-regulated at early time points and is putatively involved in dampening the immune response [35]. In this study, the gene was rapidly up-regulated by trimers at 1, 3 and 6 h, similarly to the long-OG mix, indicating that both short and long OGs trigger this gene [35].

*PI-LTP*, a lipid transfer protein, was up-regulated by the trimers at 3 and 6 h and by the long-OG mix from 1 to 24 h. Lipid transfer proteins have been identified as antimicrobial peptides and significant contributors to plan defense [65].

In conclusion, the qRT-PCR expression analysis of genes associated with the activation of OG-triggered DAMP responses or those considered markers for phytohormone signaling revealed that the plant response to trimers and longer OGs is overall quite similar, especially at the earlier time points. While there seems to be a difference in the amplitude of gene expression, i.e. plant treatment with the long-OG mix seems to result in stronger induction, the trimers are nevertheless also potent elicitors of genes involved in defense.

### Trimers elicit plant defense responses leading to enhanced resistance to infection by *P. carotovorum*

**Fig. 6:**
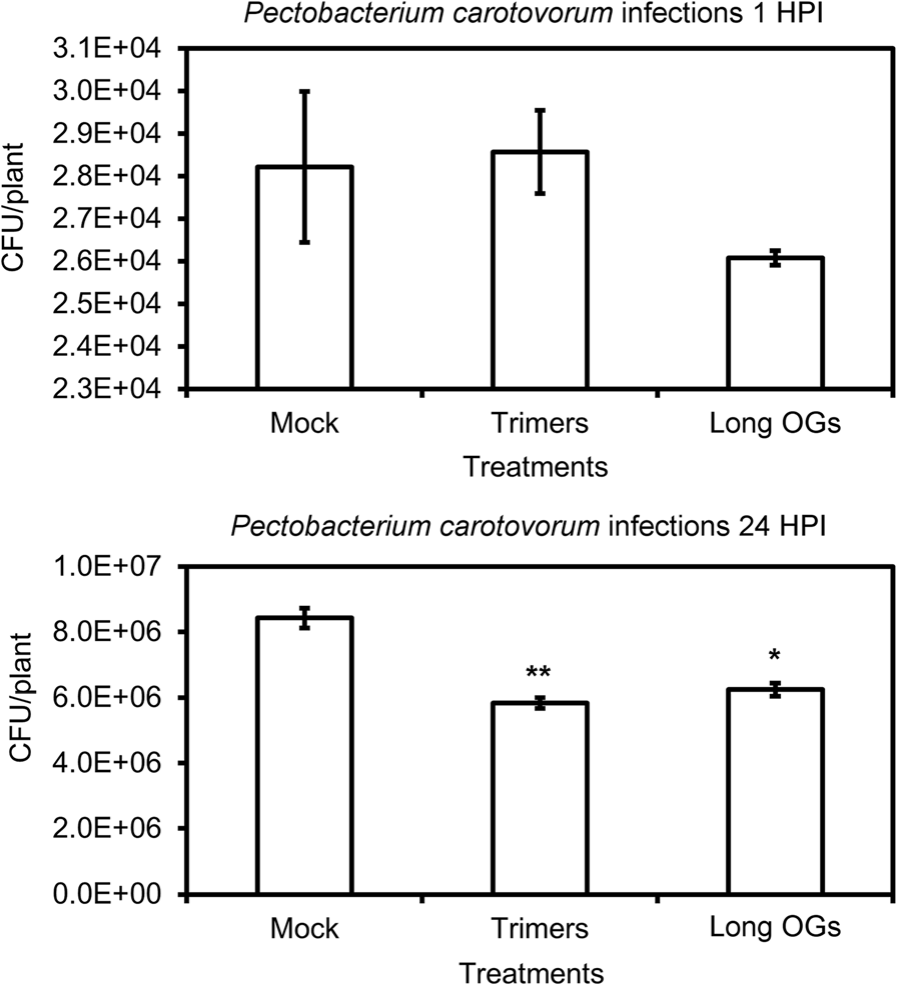
Treatment with trimers improves resistance to the necrotrophic pathogen *P. carotovorum* in Arabidopsis. Virulence assays were performed on in vitro-grown plants. Plants were pretreated by pipetting 2 μl of MQ water (0.025% Silwet), trimers (20 μM, 0.025% Silwet), or the long-OG mix (DP > 8, 20 μM, 0.025% Silwet). Subsequently, the treated plants were inoculated with *P. carotovorum* by flooding. Bacterial counts of *P. carotovorum* were performed 1 and 24 h post-infection (HPI). The difference between trimers or long-OG mix and mock treatment at 24 HPI was considered significant using Student’s *t*-test (*P* < 0.05) marked with an *asterisk* (1 asterisk = *P* < 0.05 and 2 asterisks = *P* < 0.01). The error bars in the diagrams indicate the standard error of the mean

The perception of a phytopathogen triggers the synthesis and accumulation of specific phytohormones that mediate associated defense signaling. In Arabidopsis, resistance to necrotrophic pathogens, such as *B. cinerea* and *P. carotovorum,* is affected by the phytohormones JA, ET and SA [13, 66, 67]. Because our RNA sequencing analysis indicated that JA/ET-related signaling is induced in response to trimers, we sought to determine whether pretreatment with this compound could elicit plant resistance to subsequent infection by a necrotrophic pathogen*.* We treated in vitro plants with trimers (20 μM and 0.025% Silwet) or long OGs (20 μM and 0.025% Silwet) for 24 h before inoculation with *P. carotovorum*. Compared to mock-treated (MQ and 0.025% Silwet) plants, there was a significant reduction of bacterial growth 24 h post-inoculation in plants treated with the trimers and long OGs, but no significant difference between the different OGs (Fig. 6).

This clearly indicates that trimers can prime plant defenses to *P. carotovorum*.

### Trimers inhibit growth in Arabidopsis

**Fig. 7:**
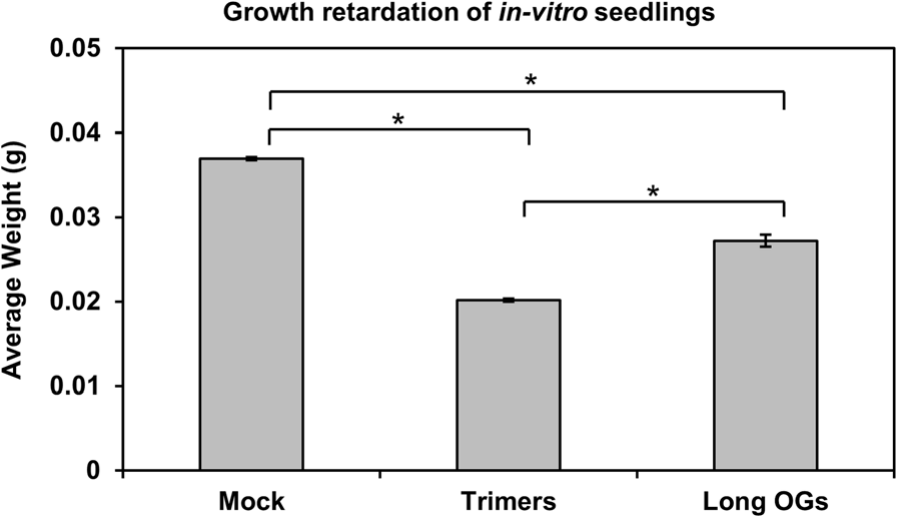
Trimers and the long-OG mix (DP > 8) induce growth retardation in Arabidopsis. Each biological replicate is a pool of four plants grown in individual wells on a 12-well plate. At least six biological replicates were used for each treatment. Differences between the trimers (200 μM), long-OG mix (200 μM) and mock (1/2 MS) treatments were statistically significant for *P* < 0.05 using ANOVA and Scheffe’s *post hoc* test, indicated by asterisks in the figure. The data shown represent three replicate experiments with similar outcomes. The error bars indicate the standard error

The activation of plant defenses diverts resources from plant growth [68]. Consistently, our transcriptomic data indicate that GO-based gene sets related to plant growth, organelle organization, cellular component biogenesis and photosynthesis were down-regulated in plants treated with trimers (Additional file 3: Table S4). To determine whether trimers were indeed capable of inhibiting growth, Arabidopsis plants were grown in vitro in medium containing trimers (200 μM), long-OG mix (DP > 8, 200 μM), or Mock. Arabidopsis seedlings treated with either of the OG preparations displayed significant growth retardation (Fig. 7). However, treatment with trimers produced growth retardation to a significantly greater extent than treatment with the long-OG mix.

### Long OGs trigger a ROS burst, whereas trimers do not

**Fig. 8:**
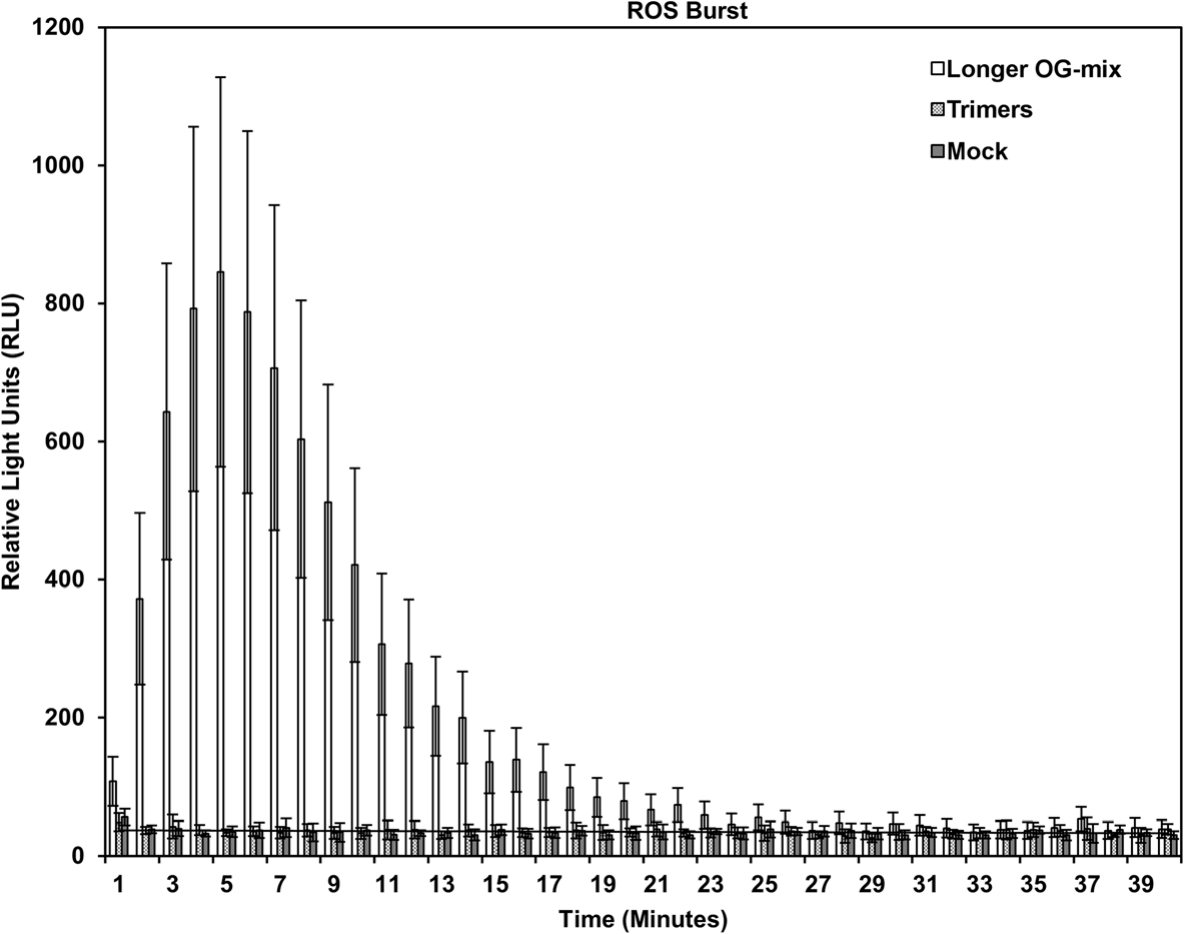
The long-OG mix (DP > 8) induce a rapid ROS burst in leaf discs, whereas trimers do not. Reactive oxygen species (ROS) burst in RLU as measured by EnSpire® Multimode Plate Reader for leaf discs exposed to the long-OG mix (200 μM), trimers (200 μM) or mock (MQ water). The error bars depict the standard deviation at each time point

Plant defense activation by long OGs and other elicitors in Arabidopsis is typically associated with the induction of a ROS burst from the plasma membrane NADPH oxidase AtRBOHD [64]. The induction of *AtRBOHD* has previously been observed upon treatment with long OGs [17, 30, 35]. Here, the transcriptomic data show a transient induction also after treatment with trimers (Fig. 5). To determine whether short OGs could also induce an initial ROS burst, we measured the early ROS induced by treating plants with trimers. In accordance with previous data [69], only the long-OG mix (DP > 8) elicited a ROS burst significantly more intense than that of the mock treatment at early time points (Fig. 8). Even higher concentrations (up to 10 mM) of trimers did not elicit a ROS burst (data not shown). However, the At*RBOHD*-mediated oxidative burst elicited by longer OGs is not required for the expression of several genes typically considered to be OG-responsive, nor for OG-induced resistance to *B. cinerea* [52]. Thus, while trimers do not induce a detectable oxidative burst in plants, this does not necessarily diminish their role in eliciting plant defense. Furthermore, trimers were found to influence the expression of a somewhat different set of peroxidases than did long OGs (Additional file 1: Table S2), possibly effecting ROS homeostasis.

### Trimers trigger MAPK3 and MAPK6 phosphorylation in Arabidopsis seedlings

**Fig. 9:**
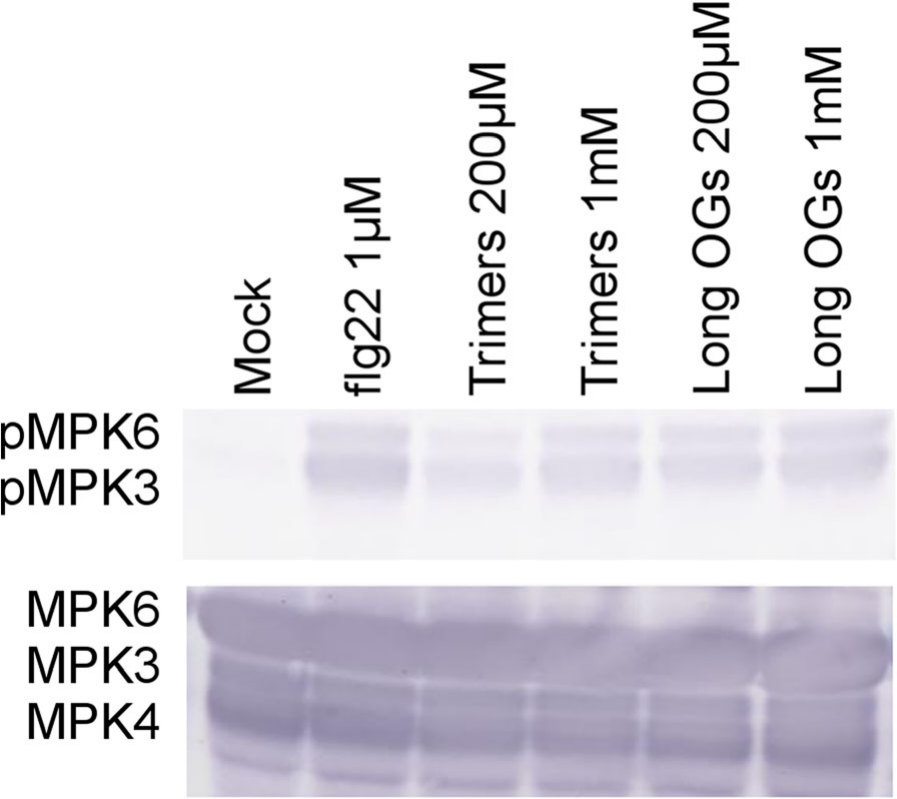
Trimers, long-OG mix (DP > 8) and flg22 induce MAPK3and MAPK6 phosphorylation. Seedlings were treated for 10 min with trimers (200 μM & 1 mM), long-OG mix (200 μM & 1 mM), flg22 (1 μM), or mock solution (1/2 MS). Each biological replicate is a pool of three wells of seedlings, each containing approximately ten plants. Thirty micrograms of each protein sample was loaded and phosphorylation levels were assessed by immunoblotting using a phospho-p44/42 specific antibody (*top*). MPK3, MPK4 and MPK6 total protein amounts were assessed using specific antibodies (*bottom*). The experiment was repeated with a second biological replicate, yielding identical results

Mitogen-activated protein kinase (MAPK) cascades participate in defense responses triggered by various elicitors, for example flg22 and OGs have been shown to cause phosphorylation of MPK3 and MPK6 [35, 70–72]. To determine whether trimers could also induce MPK3 and MPK6 phosphorylation we used immunoblotting to assay the phosphorylation levels 10 min after treatment with trimers. In accordance with previous data the long-OG mix (DP > 8) and flg22 triggered phosphorylation of MPK3 and MPK6 [71, 35]. Trimers also triggered phosphorylation, possibly somewhat less with 200 μM than 1 mM. Long OGs caused similar amount of phosphorylation independently of concentration, implying saturation of the response at 200 μM. Flg22 treatment resulted in more phosphorylation than either OG treatment (Fig. 9).

## Discussion

Oligogalacturonides (OGs) are oligomers of alpha-1,4-linked galacturonosyl residues released from plant cell walls upon pathogen attack or mechanical wounding. OGs act as damage-associated molecular patterns (DAMPs) and elicit plant defense responses similar to those of well-characterized pathogen-associated molecular patterns (PAMPs), such as the bacterial flagellin [64, 73, 74]. DAMPs, such as OGs, trigger the accumulation of reactive oxygen species (ROS), defense-associated proteins and callose, which contribute to enhanced pathogen resistance in plants [17, 52, 75, 76]. This enhanced resistance has been associated with long OGs (DP > 10) [17, 30, 35, 36]. However, there are several indications that also short OGs are potent elicitors of plant defense gene expression and may act as DAMPs to trigger plant innate immunity [28, 39, 41]. Furthermore, short OGs play a role in modulating plant development [30, 40]. To elucidate the putative functional divergence between OGs of different DPs, we explored the capacity of trimers to elicit plant defenses and to control plant growth and development. This was performed by characterizing the transcriptome using RNA sequencing from trimer-treated Arabidopsis, combined with an analysis of the physiological and phenotypic consequences of the same treatment. Furthermore, we compared the gene expression data obtained from trimer-treated Arabidopsis seedlings with data from previous microarray studies of Arabidopsis responses to long OGs [17, 30, 35]. The resulting comparative transcriptomic data reveal that trimers induce the expression of many defense-related genes in Arabidopsis. Additionally, treatment with short OGs leads to the down-regulation of genes involved in plant growth and development.

The impact of short OGs on plant defense and development was further analyzed using a GSEA. GO-based gene sets involved in stimulus (immunity and hormonal (mainly JA and ET)) responses were induced by trimers, while gene sets involved in photosynthesis, general metabolism, development and transcription were down-regulated (Figs. 4 and 5). In conclusion, RNA sequencing data from plants exposed to trimers are generally qualitatively, if not quantitatively, similar to the plant gene expression effects triggered by long OGs. Overall, the GSEA results, similar to the gene expression data, suggest that long OGs may have a greater impact on the transcriptome, but trimers still have a significant global impact.

The trimer-triggered induction of defense-associated genes and the down-regulation of genes involved in morphogenesis and development is generally consistent with the effects that long OGs induce in plants [35]. The trimers described here regulate more than 120 genes (Additional file 2: Table S3) similarly to the long OGs reported in other studies [17, 35]. A GSEA of common trimer and long-OG-regulated genes at 3 h exhibited a similar gene set map as that for trimers alone (Figs. 3 and 4).

The qRT-PCR data, together with the common genes and gene sets identified in the transcriptomic analyses as modulated in expression in response to both trimers and long OGs, indicate that both long and short OGs induce significant changes in the expression of genes involved in signaling associated with plant defense and growth. In general, while trimers and the long-OG mix induced similar expression of the corresponding marker genes, some differences in the timing and amplitude of the response were evident, particularly at the early time points.

Here, trimers triggered a stronger expression of *PGIP1-2* than that observed in response to the long-OG mix. This could reflect varying plant responses to the appearance of OGs with different DPs during infection. Trimers supposedly appear at the point when the infection has already proceeded further; therefore, the plant responds with a higher induction of *PGIP1-2* to inhibit the degrading enzymes of the pathogen.

Tissue damage, triggered for example by wounding or infection by necrotrophic pathogens, induces specific defense responses, such as the activation of glucosinolate defenses. This is accompanied with the rapid activation of JA synthesis and signaling. The fast induction of *CYP81F2* (Fig. 5) suggests that trimers are indeed prominent inducers of DAMP signaling. The clear up-regulation of the positive regulator of JA-signaling *WRKY40* further supported this notion (Fig. 5). The phytohormones auxin and ABA have a complex role in defense and growth signaling, as well as cross talk between each other [73, 77, 78]. Their activity according to marker genes such as *RAB18*, *ABR1*, *TAR2*, *NCED4* and *IAA2* was found in the RNASeq data to be ambiguous due to their contrasting up and down-regulation. However, trimers were observed to have a clearly retarding effect on Arabidopsis seedling growth (Fig. 7), which was mirrored by the down-regulation of the auxin marker gene *SAUR-AC1* according to qPCR in response to trimers mirrored this growth retardation, underlining the negative effect defense signaling has on processes associated with growth.

Short OGs have previously been linked to an inability to induce a ROS-burst in *Nicotiana tabacum* cv. Petit Havana [69]. Accordingly, while we detected a clear ROS burst in Arabidopsis leaves treated with the long-OG mix, this was not noticed for trimers. The same has been observed by Legendre et al. (1993), who showed that the induction of H_2_O_2_ accumulation requires OGs with DP > 3 in suspension-cultured soybean cells [77]. Interestingly, we observed the induction of *AtRBOH* in response to both trimers and the long-OG mix. However, the expression induced by trimers appeared to be much shorter in duration (Fig. 5). Thus, trimers and long OGs differ in their ability to induce *AtRBOHD* and to trigger the initial ROS burst in Arabidopsis. Typically the ROS burst is associated with HR-associated cell death, which is considered to promote susceptibility to necrotrophs [68]. It would be tempting to speculate that at the early stages of infection, when mostly long OGs are present, the ROS burst and subsequent signaling could be beneficial in fighting off the pathogen. At the later stages of infection, when shorter OGs are supposedly dominating, the initiation of ROS production and cell death might be detrimental. Furthermore, trimers may still contribute to altered ROS homeostasis, possibly by affecting the induction of peroxidase genes (Additional file 1: Table S2).

Our results show that plants grown in the presence of trimers or the commercial long-OG mix exhibited reduced growth (Fig. 7). Furthermore, the in vitro treatment of seedlings with trimers induced plant defense against *P. carotovorum* (Fig. 6). The resource allocation from growth- to defense-related processes results from the rapid activation of DAMP signaling in response to trimer treatment; the binding of PAMPs or DAMPs to their corresponding receptors has previously been shown to trigger defense-associated mechanisms, such as ET biosynthesis, ROS burst, callose deposition and seedling growth inhibition [78]. The pretreatment of plants with known DAMPs, such as long OGs or the protein Pep1, has been demonstrated to elicit resistance to pathogens, such as *P. syringae* or *B. cinerea* [64, 76].

Although both long and short OGs produce a significant phosphorylation response in MAPKs at micromolar concentrations, the bacterial flg22 produces significantly more phosphorylation, indicating that the maximum capacity for phosphorylation of these two proteins is not reached by OGs, or possibly that it is reached at a difference in timing. This is in accordance with what was reported by [35] who observed a stronger and longer lasting activity of MPK3 in Arabidopsis mesophyll protoplasts treated with OGs, than those treated with flg22. In conclusion, these data demonstrate that in addition to long OGs (DP > 10), also short OGs (DP3) are biologically active and capable of inducing defense gene expression, triggering defense related pathways, inhibiting seedling growth, as well as eliciting defense against the necrotrophic pathogen *P. carotovorum* in Arabidopsis.

## Conclusions

This study provides a novel characterization of the general impact of trimers on the global Arabidopsis transcriptome. The RNA sequencing data from this study were compared with data from previous studies characterizing the global transcriptomic effects of long OGs in Arabidopsis [35, 64]. From this comparison, it became clear that although trimer treatment had less of an overall impact on the transcriptome than did the long OGs at early time points, trimers constitute a biologically active DAMP signal. This conclusion is in agreement with previous studies suggesting that there is not necessarily any minimum DP limit for OG activity [39]. In future studies, it will be of interest to perform a more encompassing analysis of the distinct plant responses to OGs with specific DPs.

## Methods

### Plant material and growth conditions

All of the experiments in this study used plant material from *Arabidopsis thaliana* Columbia-0 (Col-0) ecotype (Arabidopsis), obtained by the Nottingham *A. thaliana* Stock Centre. Seeds were surface sterilized using chlorine gas [79]. Briefly, the seeds were placed in 2 ml microcentrifuge tubes inside a desiccator. A glass container with 150 ml of sodium hypochlorite (Sigma-Aldrich, 10%) and 4.2 ml of HCl (Sigma-Aldrich, 37%) was placed in the desiccator. The lid was immediately sealed with tape, and the seeds were left for 68 h before the gas was allowed to dissipate. The plants used in virulence assays were grown in a growth room in pots containing a 1:1 mixture of soil and peat. The light period was 12 h, and the temperature was 22 °C. After 3–4 weeks, the plants were used for physiological assays.

### Analysis of oligogalacturonide DP

Commercially available trimers (Trigalacturonic acid, Sigma-Aldrich) and a long-OG mix with a stated DP10-15 (Elicityl) were run on Silica gel 60_F254_ TLC plates and analyzed by mass spectrometry to confirm the degree of polymerization. Trimers were found to consist of only DP3, whereas the long-OG mix was found to consist of primarily DP8-19, with an average DP of approximately 14 (Additional file 10: Figure S3, Additional file 11: Figure S4 and Additional file 12: Figure S5).

### Growth inhibition assay

For the growth inhibition assay, Arabidopsis seedlings were placed in individual wells with 1 ml of liquid 1/2 MS medium in 12-well plates [80]. The plates were sealed with Parafilm to minimize evaporation. Seeds were stratified for 3 days then allowed to grow in a 12 h light cycle at 20 °C. After 8 days, the medium was replaced with fresh liquid 1/2 MS, and the seedlings were grown for two more days before treatment with OG-containing suspensions of either trimers (200 μM) or the commercial long-OG mix (200 μM). As a mock treatment, 1/2 MS medium was used. After treatment, the plants grew for 8 days before the total plant weight was assessed. For all of the subsequent assays, 4 plants were combined into one biological replicate, and 12 biological replicates were tested per treatment. All of the experiments were performed a minimum of three times. The average plant weight per treatment was compared and statistically analyzed using an ANOVA combined with Scheffe’s *post hoc* test.

### RNA extraction and purification

Plant seedlings were grown in vitro*,* as described by Ferrari et al. (2007) except that the growth medium base that was used in this study was 1/2 MS and the photoperiod was 12 h [17]. Additionally, higher equimolar concentrations of trimers were used compared to those used in previously studies using long OGs [17, 35]. The concentrations used in this study were primarily set such that a significant induction of growth retardation and induction of defense genes could be observed. Briefly, the protocol for treating plants was similar to that used in growth inhibition studies except that 12 seeds were placed in each well for germination. The seedlings were treated with trimers (200 μM), the long-OG mix (200 μM) or mock solution (1/2 MS) for 3 h prior to RNA extraction. For each biological replicate, plants were harvested from 3 wells, rinsed in MQ water and blotted dry before freezing in liquid nitrogen. The plant material was crushed using metal beads in a shaker. The RNA was extracted using the GeneJET Plant RNA Purification mini kit (ThermoScientific) following the manufacturer’s protocol. The sample quality was assessed by gel electrophoresis and measured using a NanoDrop (ThermoScientific). Then, 2 μg of total RNA was treated with DNaseI and RNAse-free (ThermoScientific) and used for cDNA synthesis using Maxima Reverse Transcriptase and Ribolock RNase Inhibitor (ThermoScientific) following the manufacturer’s protocol. The primers used for cDNA synthesis were random hexamers and oligo dT primers.

### RNA Sequencing

The total RNA extracted from 3 biological replicates per treatment (trimers and mock) was purified from rRNA using an Epicenter Ribo-Zero rRNA removal kit (Plant leaf) according to the manufacturer’s instructions. The quality and quantity of the extracted RNA were assayed with a Qubit fluorometer (Life Technologies) and a NanoDrop spectrophotometer before sequencing. Sequencing was performed on the SOLiD 5500XL platform. Each sample was part of a pool and run in two separate lanes, generating approximately 9 million single-end reads with a length of 75 bp that were mapped to the genome using the SHRiMP platform [81]. A total of 33,597 gene sequences were used to align reads from sequencing. Gene expression was considered to be significantly different for FDR values <0.05 and a log_2_ fold change of ≥0.5 or ≤ -0.5 as calculated by the R package DESeq2 [82]. The raw RNA sequencing data can be found in the NCBI GEO repository under the accession number GSE69538.

### Quantitative RT-PCR analysis

Total RNA was extracted from plants that were treated with the trimers, long-OG mix or mock solution and were raised similarly to plants used for RNA sequencing. RNA for qRT-PCR analysis was extracted 1, 3, 7, 12 and 24 h post-treatment. For each time point, three biological replicates per treatment were used to generate RNA samples. For qPCR, HOT FIREPol® EvaGreen® qPCR Mix Plus (Solis Biodyne) was used with an estimated 8 ng of cDNA in a total reaction volume of 10 μl. The amplification program used on the BioRad CFX is as follows: an initial activation cycle of 95 °C for 15 min, followed by 40 cycles at 95 °C for 15 s, 60 °C for 20 s and 72 °C for 20 s, ending with a melt curve analysis. The resulting qRT-PCR data were analyzed using GeNorm software [83]. Housekeeping genes used as references in the qRT-PCR analysis were AT4G05320 (*UBQ10*), AT5G09810 (*ACT7*), AT5G12250 (*TUB6*), AT5G60390 (*EF1α*) and AT3G13920 (*eIF4A*). These genes were assessed for stability across cDNA samples using the GeNorm M-value principle in the GenEx 6 software (MultiD Analyses AB, Gothenburg, Sweden). The two most stable reference genes (*EF1α* and *eIF4A*) had an M-value <0.3 across all samples and were used for the subsequent normalization [83, 84]. All of the analyzed genes, along with their respective primers, are listed in Additional file 13: Table S1. Statistically significant differences between treatments were determined by Student’s *t*-test comparing dCt values.

### Infection assays

In vitro plant infections were performed by the flood-inoculation method [85]. *A. thaliana* seedlings were grown under axenic conditions on 1/2 MS [86] agar plates for 10-12 days (12 h day/night cycle at 20 °C) prior to pretreatment. The plants were pre-treated with the application of a 2 μl drop of either sterile MQ water (0.025% Silwet), trimers (20 μM, 0.025% Silwet), or the long-OG mix (20 μM, 0.025% Silwet) to two rosette leaves per plant approximately 24 h before infection. *Pectobacterium carotovorum* sp. *carotovorum* SCC1 was cultured overnight in liquid Luria-Bertani (LB) medium at 28 °C. Bacterial cultures were washed twice in 10 mM MgSO_4_ and resuspended in 35 ml of a solution containing 10 mM MgSO_4_ and 0.025% silwet. The OD_600_ of the bacterial suspension was adjusted to 0.001 before flooding the *A. thaliana* seedlings for approximately 2.5–3 min. After the bacterial suspension was decanted, the plants were incubated overnight under conditions consistent with those before inoculation. Each plate contained 6 plants that were combined to produce one biological replicate. Plants were crushed, and the resulting liquid was diluted and spotted onto LB plates. For each dilution and sample, five drops of 20 μl each were plated for bacterial counting. Four biological replicates were used for each treatment at the 2 h time point, and 12 biological replicates were used for the 24 h time point. The experiment was repeated with similar results. Statistical significance was calculated using a Student’s *t*-test (*P* < 0.01).

### Quantification of ROS production

Prior to treatment, leaf discs (approximately 2 mm in diameter) were floated on MQ water for 24 h in 96-well plates. Next, the water was replaced with 100 μl of a solution containing 20 mM luminol, 10 μg/ml peroxidase and either MQ water, trimers (200, 400 or 800 μM) or the long-OG mix (200 μM). The relative light units (RLUs) produced in each well were measured every minute for 40 min using an EnSpire® Multimode Plate Reader [87].

### Bioinformatics analysis

RNA sequencing data from plants exposed to short OGs, together with previously published microarray data from plants treated with long OGs (using a *P*-value of <0.01 as a cut-off, as in the corresponding studies), were analyzed for gene set enrichment using the GenePattern software (Broad Institute). Software parameters included 1000 permutations and a gene set size range from 5 to 1000 [17, 35, 88, 89]. A *P*-value cut-off of <0.05 was used to determine gene sets with significantly altered expression. Furthermore, a Kyoto Encyclopedia of Genes and Genomes (KEGG, http://www.genome.jp/kegg/)-based pathway analysis was performed using the R package Pathview [90].

### Total protein extraction

Plant seedlings were grown as those used for RNA extraction. The seedlings were treated with trimers (200 μM and 1 mM), the long-OG mix (200 μM and 1 mM), flg22 (1 μM), or mock solution (1/2 MS) for 10 min prior to protein extraction. For each biological replicate, plants were harvested from 3 wells, rinsed in MQ water and blotted dry before freezing in liquid nitrogen. The plant material was crushed using metal beads in a shaker. Total protein was extracted in extraction buffer (50 mM Tris-Hcl pH 7.5, 200 mM NaCl, 1 mM EDTA, 10% Glycerol, 1% Triton X-100, 1 mM Pefabloc, 1 mM DTT, 1 x Protease inhibitor (Roche Diagnostics) and 1 x Phosphatase inhibitor (Thermo Fisher Scientific)), incubated on ice for 30 min and then centrifuged for 15 min at 21 500 g at 4 °C.

### MPK3 and MPK6 immunoblotting

Immunodetection was performed similarly to Ortiz-Morea et al. 2016 [91]. Briefly, 30 μg of protein from each sample was boiled for 3 min in Laemmli buffer, resolved on a 12% polyacrylamide gels before being transferred onto a PVDF membrane (Immobilion). The membrane was blocked for 2 h with 5% BSA before being incubated with primary antibodies (MPK3-Sigma M8318 Anti-AtMPK3 antibody produced in rabbit 1:1000, MPK6-Sigma A7104 Anti-AtMPK6 antibody produced in rabbit 1:3000, MPK4-Sigma A6979 Anti-AtMPK4 antibody produced in rabbit 1:1500, or Phospho-p44/42 MAPK-(Erk1/2) (Thr202/Tyr204) Antibody #9101 Cell Signaling Technology. 1:1000) overnight. Incubation with secondary antibody (Anti-Rabbit IgG Alkaline Phosphatase Conjugate-Promega 1:10 000) was done for 1 h. Visualization was performed using the AP Conjugate Substrate Kit (Bio-Rad Laboratories).

## Additional files

Additional file 1: Table S2. RNA-sequencing gene expression data of plants treated by trimers compared to plants treated by a mock. Corresponding data is presented from plants treated with microarray results from long OG treatment at 1 h (long OGs 1 h - Denoux et al. 2008), 3 h (long OGs 3 h - Denoux et al. 2008) and 2 h (long OGs 2 h, cell culture - Moscatiello et al. 2006). #N/A = not significantly affected in expression. (XLSX 242 kb)
Additional file 2: Table S3. Genes significantly affected in expression (Fold Change) by treatment with trimers at 3 h (Trimers), long OGs at 1 h in the study by Denoux et al. 2008 (Long OGs 1 h) and long OGs at 3 h in the study by Denoux et al. 2008 (Long OGs 3 h). (XLSX 51 kb)
Additional file 3: Table S4. GSEA results based on gene expression data from trimer treated plants compared to mock treatment. (XLSX 31 kb)
Additional file 4: Table S5. GSEA result of genes affected similarly in expression by both trimers and long OGs at 3 h (Denoux et al. 2008). (XLSX 36 kb)
Additional file 5: Table S6. GSEA result of genes affected in expression by both Trimers (3 h) and long OGs (1 h and 3 h-Denoux et al. 2008). (XLSX 26 kb)
Additional file 6: Table S7. GSEA results based on gene expression data from trimer treated plants, disregarding genesets also affected by long OGs at 3 h (Denoux et al. 2008). (XLSX 11 kb)
Additional file 7: Table S8. GSEA results based on gene expression data of long OGs at 3 h (Denoux et al. 2008) disregarding genesets also affected by trimers. (XLSX 41 kb)
Additional file 8: Figure S1. Pathway analysis of plant hormone signal transduction pathways using RNA-sequencing data of plants treated by trimers compared to plants treated by a mock (Trimers). Also used is corresponding data from plants treated by long OGs at 1 h (long OGs 1 h) and 3 h (long OGs 3 h) (Denoux et al. 2008). (TIF 1568 kb)
Additional file 9: Figure S2. Pathway analysis of plant-pathogen interaction pathways using RNA-sequencing data of plants treated by trimers compared to plants treated by a mock (Trimers). Also used is corresponding data from plants treated by long OGs at 1 h (long OGs 1 h) and 3 h (long OGs 3 h) (Denoux et al. 2008). (TIF 2399 kb)
Additional file 10: Figure S3. Mass spectrum graph for the trimeric OGs used in this study (Trimers). (TIF 759 kb)
Additional file 11: Figure S4. Mass spectrum graph for the long OGs with a degree of polymerization >8 used in this study (long-OG mix). (TIF 1425 kb)
Additional file 12: Figure S5. Results of TLC plate analysis of Trimers and long-OG mix (longer OG mix). (TIF 973 kb)
Additional file 13: Table S1. The qRT-PCR primers (nucleotide sequences) used in this study, their target gene ID, gene name and applicable references. (XLSX 11 kb)

## Abbreviations

ABA: Abscisic Acid
DAMP: Damage Associated Molecular Pattern
DP: Degree of Polymerization
ET: Ethylene
GO: Gene Ontology
GSEA: GeneSet Enrichment Analysis
JA: Jasmonic Acid
OG: Oligogalacturonides
PAMP: Pathogen Associated Molecular Pattern
PCWDE: Plant Cell Wall-Degrading Enzyme
qRT-PCR: Quantitative RT-PCR
ROS: Reactive Oxygen Species
SA: Salicylic Acid
Trimers: Trigalacturonic acid

## References

[1] Charkowski A, Blanco C, Condemine G, Expert D, Franza T, Hayes C (2012). The role of secretion systems and small molecules in soft-Rot Enterobacteriaceae pathogenicity. Annu Rev Phytopathol.

[2] Liu H, Coulthurst SJ, Pritchard L, Hedley PE, Ravensdale M, Humphris S (2008). Quorum sensing coordinates brute force and stealth modes of infection in the plant pathogen pectobacterium atrosepticum. PLoS Pathog.

[3] Wang X, Jiang N, Liu J, Liu W, Wang G-L (2014). The role of effectors and host immunity in plant–necrotrophic fungal interactions. Virulence.

[4] Lai Z, Mengiste T (2013). Genetic and cellular mechanisms regulating plant responses to necrotrophic pathogens. Curr Opin Plant Biol.

[5] Ma B, Hibbing ME, Kim H-S, Reedy RM, Yedidia I, Breuer J (2007). Host range and molecular phylogenies of the soft Rot enterobacterial genera pectobacterium and dickeya. Phytopathology.

[6] Mansfield J, Genin S, Magori S, Citovsky V, Sriariyanum M, Ronald P (2012). Top ten plant pathogenic bacteria in molecular plant pathology. Mol Plant Pathol.

[7] Collmer A, Schneider DJ, Lindeberg M (2009). Lifestyles of the effector rich: genome-enabled characterization of bacterial plant pathogens. Plant Physiol.

[8] Toth IK, Pritchard L, Birch PRJ (2006). Comparative genomics reveals what makes an enterobacterial plant pathogen. Annu Rev Phytopathol.

[9] Toth IK, Birch PR (2005). Rotting softly and stealthily. Curr Opin Plant Biol.

[10] Davidsson PR, Kariola T, Niemi O, Palva T (2013). Pathogenicity of and plant immunity to soft rot pectobacteria. Plant Microbe Interact.

[11] Kariola T, Brader G, Li J, Palva ET (2005). Chlorophyllase 1, a damage control enzyme, affects the balance between defense pathways in plants. Plant Cell Online.

[12] Li J, Brader G, Palva ET (2004). The WRKY70 transcription factor: a node of convergence for jasmonate-mediated and salicylate-mediated signals in plant defense. Plant Cell Online.

[13] Norman-Setterblad C, Vidal S, Palva ET (2000). Interacting signal pathways control defense gene expression in Arabidopsis in response to cell wall-degrading enzymes from Erwinia carotovora. Mol Plant Microbe Interact.

[14] Palva TK, Hurtiga M, Saindrenan P, Palva ET (1994). Salicylic acid induced resistance to Erwinia carotovora subsp. Carotovora in tobacco. Mol Plant Microbe Interact.

[15] Vidal S, de León IP, Denecke J, Palva ET (1997). Salicylic acid and the plant pathogen Erwinia carotovora induce defense genes via antagonistic pathways. Plant J.

[16] Bolton MD, Thomma BPHJ, Nelson BD (2006). Sclerotinia sclerotiorum (Lib.) de Bary: biology and molecular traits of a cosmopolitan pathogen. Mol Plant Pathol.

[17] Ferrari S, Galletti R, Denoux C, Lorenzo GD, Ausubel FM, Dewdney J (2007). Resistance to botrytis cinerea induced in Arabidopsis by elicitors is independent of salicylic acid, ethylene, or jasmonate signaling but requires PHYTOALEXIN DEFICIENT3. Plant Physiol.

[18] Gravino M, Savatin DV, Macone A, De Lorenzo G (2015). Ethylene production in *Botrytis cinerea-* and oligogalacturonide-induced immunity requires calcium-dependent protein kinases. Plant J.

[19] Broberg M, Lee GW, Nykyri J, Lee YH, Pirhonen M, Palva ET. The global response regulator ExpA controls virulence gene expression through RsmA-mediated and RsmA-independent pathways in Pectobacterium wasabiae SCC3193. Appl Environ Microbiol. 2014. 10.1128/AEM.03829-13 PMC3957638 24441162

[20] Alfano JR, Collmer A (1996). Bacterial pathogens in plants: life up against the wall. Plant Cell Online.

[21] Abbott DW, Boraston AB (2008). Structural biology of pectin degradation by Enterobacteriaceae. Microbiol Mol Biol Rev.

[22] Micheli F (2001). Pectin methylesterases: cell wall enzymes with important roles in plant physiology. Trends Plant Sci.

[23] Barras F, van Gijsegem F, Chatterjee AK (1994). Extracellular enzymes and pathogenesis of soft-Rot Erwinia. Annu Rev Phytopathol.

[24] Shah P, Gutierrez-Sanchez G, Orlando R, Bergmann C (2009). A proteomic study of pectin-degrading enzymes secreted by botrytis cinerea grown in liquid culture. Proteomics.

[25] Bishop PD, Makus DJ, Pearce G, Ryan CA (1981). Proteinase inhibitor-inducing factor activity in tomato leaves resides in oligosaccharides enzymically released from cell walls. Proc Natl Acad Sci.

[26] Campbell AD, Labavitch JM (1991). Induction and regulation of ethylene biosynthesis by pectic oligomers in cultured pear cells. Plant Physiol.

[27] Hahn MG, Darvill AG, Albersheim P, Host-Pathogen Interactions XIX (1981). The endogenous elicitor, a fragment of a plant cell wall polysaccharide that elicits phytoalexin accumulation in soybeans. Plant Physiol.

[28] Norman C, Vidal S, Palva ET (1999). Oligogalacturonide-mediated induction of a gene involved in jasmonic acid synthesis in response to the cell-wall-degrading enzymes of the plant pathogen Erwinia carotovora. Mol Plant Microbe Interact.

[29] Ridley BL, O’Neill MA, Mohnen D (2001). Pectins: structure, biosynthesis, and oligogalacturonide-related signaling. Phytochemistry.

[30] Moscatiello R, Mariani P, Sanders D, Maathuis FJM (2006). Transcriptional analysis of calcium-dependent and calcium-independent signalling pathways induced by oligogalacturonides. J Exp Bot.

[31] Côté F, Hahn MG, Palme K (1994). Signals signal transduct. Pathw. Plants [internet].

[32] Doares SH, Syrovets T, Weiler EW, Ryan CA (1995). Oligogalacturonides and chitosan activate plant defensive genes through the octadecanoid pathway. Proc Natl Acad Sci U S A.

[33] Ellis C, Karafyllidis I, Wasternack C, Turner JG (2002). The Arabidopsis mutant cev1 links cell wall signaling to jasmonate and ethylene responses. Plant Cell Online.

[34] Sharrock KR, Labavitch JM (1994). Polygalacturonase inhibitors of Bartlett pear fruits: differential effects on botrytis cinerea polygalacturonase isozymes, and influence on products of fungal hydrolysis of pear cell walls and on ethylene induction in cell culture. Physiol Mol Plant Pathol.

[35] Denoux C, Galletti R, Mammarella N, Gopalan S, Werck D, Lorenzo GD (2008). Activation of defense response pathways by OGs and Flg22 elicitors in Arabidopsis seedlings. Mol Plant.

[36] Federici L, Di Matteo A, Fernandez-Recio J, Tsernoglou D, Cervone F (2006). Polygalacturonase inhibiting proteins: players in plant innate immunity?. Trends Plant Sci.

[37] Brutus A, Sicilia F, Macone A, Cervone F, Lorenzo GD (2010). A domain swap approach reveals a role of the plant wall-associated kinase 1 (WAK1) as a receptor of oligogalacturonides. Proc Natl Acad Sci.

[38] Decreux A, Thomas A, Spies B, Brasseur R, Cutsem PV, Messiaen J (2006). In vitro characterization of the homogalacturonan-binding domain of the wall-associated kinase WAK1 using site-directed mutagenesis. Phytochemistry.

[39] Simpson SD, Ashford DA, Harvey DJ, Bowles DJ (1998). Short chain oligogalacturonides induce ethylene production and expression of the gene encoding aminocyclopropane 1-carboxylic acid oxidase in tomato plants. Glycobiology.

[40] Miranda JH, Williams RW, Kerven G (2007). Galacturonic acid-induced changes in strawberry plant development in vitro. Vitro cell. Dev Biol Plant.

[41] Weber J, Olsen O, Wegener C, von Wettstein D (1996). Digalacturonates from pectin degradation induce tissue responses against potato soft rot. Physiol Mol Plant Pathol.

[42] Thain JF, Doherty HM, Bowles DJ, Wildon DC (1990). Oligosaccharides that induce proteinase inhibitor activity in tomato plants cause depolarization of tomato leaf cells. Plant Cell Environ.

[43] Montesano M, Kõiv V, Mäe A, Palva ET (2001). Novel receptor-like protein kinases induced by Erwinia carotovora and short oligogalacturonides in potato. Mol Plant Pathol.

[44] Moloshok T, Pearce G, Ryan CA (1992). Oligouronide signaling of proteinase inhibitor genes in plants: structure-activity relationships of Di- and trigalacturonic acids and their derivatives. Arch Biochem Biophys.

[45] Pontiggia D, Ciarcianelli J, Salvi G, Cervone F, De Lorenzo G, Mattei B. Sensitive detection and measurement of oligogalacturonides in Arabidopsis. Front Plant Sci [Internet]. 2015 [cited 2016 Oct 27];6. Available from: http://journal.frontiersin.org/article/10.3389/fpls.2015.00258/abstract 10.3389/fpls.2015.00258 PMC4404744 25954288

[46] An HJ, Lurie S, Greve LC, Rosenquist D, Kirmiz C, Labavitch JM (2005). Determination of pathogen-related enzyme action by mass spectrometry analysis of pectin breakdown products of plant cell walls. Anal Biochem.

[47] Roy C, Kester H, Visser J, Shevchik V, Hugouvieux-Cotte-Pattat N, Robert-Baudouy J (1999). Modes of action of five different endopectate lyases from Erwinia chrysanthemi 3937. J Bacteriol.

[48] Fagard M, Dellagi A, Roux C, Périno C, Rigault M, Boucher V (2007). Arabidopsis thaliana expresses multiple lines of defense to counterattack Erwinia chrysanthemi. Mol Plant Microbe Interact.

[49] Forrest RS, Lyon GD (1990). Substrate degradation patterns of polygalacturonic acid lyase from Erwinia carotovora and bacillus polymyxa and release of phytoalexin-eliciting oligosaccharides from potato cell walls. J Exp Bot.

[50] Preston JF, Rice JD, Ingram LO, Keen NT (1992). Differential depolymerization mechanisms of pectate lyases secreted by Erwinia chrysanthemi EC16. J Bacteriol.

[51] Bartling S, Wegener C, Olsen O (1995). Synergism between Erwinia pectate lyase isoenzymes that depolymerize both pectate and pectin. Microbiol Read Engl.

[52] Galletti R, Denoux C, Gambetta S, Dewdney J, Ausubel FM, Lorenzo GD (2008). The AtrbohD-mediated oxidative burst elicited by oligogalacturonides in Arabidopsis is dispensable for the activation of defense responses effective against botrytis cinerea. Plant Physiol.

[53] Yi X, Du Z, Su Z (2013). PlantGSEA: a gene set enrichment analysis toolkit for plant community. Nucleic Acids Res.

[54] De Vos M, Jander G (2009). Myzus persicae (green peach aphid) salivary components induce defence responses in Arabidopsis thaliana. Plant Cell Environ.

[55] Ehlting J, Chowrira SG, Mattheus N, Aeschliman DS, Arimura G-I, Bohlmann J (2008). Comparative transcriptome analysis of Arabidopsis thaliana infested by diamond back moth (Plutella xylostella) larvae reveals signatures of stress response, secondary metabolism, and signalling. BMC Genomics.

[56] Raffaele S, Vailleau F, Léger A, Joubès J, Miersch O, Huard C (2008). A MYB transcription factor regulates very-long-chain fatty acid biosynthesis for activation of the hypersensitive cell death response in Arabidopsis. Plant Cell.

[57] Brader G, Mikkelsen MD, Halkier BA, Tapio PE (2006). Altering glucosinolate profiles modulates disease resistance in plants. Plant J Cell Mol Biol.

[58] Johnston DJ, Williamson B, McMillan GP (1994). The interaction *in planta* of polygalacturonases from *Botrytis cinerea* with a cell wall-bound polygalacturonase-inhibiting protein (PGIP) in raspberry fruits. J Exp Bot.

[59] Cabanne C, Donèche B (2002). Purification and characterization of two isozymes of polygalacturonase from botrytis cinerea. Effect of calcium ions on polygalacturonase activity. Microbiol Res.

[60] Rasul S, Dubreuil-Maurizi C, Lamotte O, Koen E, Poinssot B, Alcaraz G (2012). Nitric oxide production mediates oligogalacturonide-triggered immunity and resistance to Botrytis cinerea in Arabidopsis thaliana. Plant Cell Environ.

[61] Savatin DV, Gramegna G, Modesti V, Cervone F. Wounding in the plant tissue: the defense of a dangerous passage. Front Plant Sci. [Internet]. 2014 [cited 2016 Mar 7];5. Available from: http://www.ncbi.nlm.nih.gov/pmc/articles/PMC4165286/10.3389/fpls.2014.00470 PMC4165286 25278948

[62] Lång V, Palva ET (1992). The expression of a rab-related gene, rab18, is induced by abscisic acid during the cold acclimation process of Arabidopsis thaliana (L.) heynh. Plant Mol Biol.

[63] Pandey GK, Grant JJ, Cheong YH, Kim BG, Li L, Luan S (2005). ABR1, an APETALA2-domain transcription factor that functions as a repressor of ABA response in Arabidopsis. Plant Physiol.

[64] Ferrari S, Savatin DV, Sicilia F, Gramegna G, Cervone F, Lorenzo GD. Oligogalacturonides: plant damage-associated molecular patterns and regulators of growth and development. Front Plant Sci. [Internet]. 2013 [cited 2014 Sep 10];4. Available from: http://www.ncbi.nlm.nih.gov/pmc/articles/PMC3595604/10.3389/fpls.2013.00049 PMC3595604 23493833

[65] García-Olmedo F, Molina A, Alamillo JM, Rodríguez-Palenzuéla P (1998). Plant defense peptides. Biopolymers.

[66] Anderson JP, Badruzsaufari E, Schenk PM, Manners JM, Desmond OJ, Ehlert C (2004). Antagonistic interaction between abscisic acid and jasmonate-ethylene signaling pathways modulates defense gene expression and disease resistance in Arabidopsis. Plant Cell Online.

[67] Thomma BP, Penninckx IA, Cammue BP, Broekaert WF (2001). The complexity of disease signaling in Arabidopsis. Curr Opin Immunol.

[68] Bolton MD (2009). Primary metabolism and plant defense-fuel for the fire. Mol Plant Microbe Interact MPMI.

[69] Bellincampi D, Dipierro N, Salvi G, Cervone F, Lorenzo GD (2000). Extracellular H2O2 induced by oligogalacturonides is Not involved in the inhibition of the auxin-regulated rolB gene expression in tobacco leaf explants. Plant Physiol.

[70] Galletti R, Ferrari S, De Lorenzo G (2011). Arabidopsis MPK3 and MPK6 play different roles in basal and oligogalacturonide- or flagellin-induced resistance against Botrytis cinerea. Plant Physiol.

[71] Mattei B, Spinelli F, Pontiggia D, De Lorenzo G (2016). Comprehensive analysis of the membrane phosphoproteome regulated by oligogalacturonides in Arabidopsis thaliana. Front Plant Sci.

[72] Rasmussen MW, Roux M, Petersen M, Mundy J. MAP Kinase Cascades in Arabidopsis Innate Immunity. Front Plant Sci. [Internet]. 2012 [cited 2016 Oct 29];3. Available from: http://journal.frontiersin.org/article/10.3389/fpls.2012.00169/abstract 10.3389/fpls.2012.00169 PMC3402898 22837762

[73] Desaki Y, Otomo I, Kobayashi D, Jikumaru Y, Kamiya Y, Venkatesh B (2012). Positive crosstalk of MAMP signaling pathways in rice cells. PLoS One.

[74] Tanaka K, Choi J, Cao Y, Stacey G. Extracellular ATP acts as a damage-associated molecular pattern (DAMP) signal in plants. Front Plant Sci. [Internet]. 2014 [cited 2014 Dec 28];5. Available from: http://www.ncbi.nlm.nih.gov/pmc/articles/PMC4153020/10.3389/fpls.2014.00446 PMC4153020 25232361

[75] Krol E, Mentzel T, Chinchilla D, Boller T, Felix G, Kemmerling B (2010). Perception of the Arabidopsis danger signal peptide 1 involves the pattern recognition receptor AtPEPR1 and its close homologue AtPEPR2. J Biol Chem.

[76] Yamaguchi Y, Huffaker A, Bryan AC, Tax FE, Ryan CA (2010). PEPR2 is a second receptor for the Pep1 and Pep2 peptides and contributes to defense responses in Arabidopsis. Plant Cell Online.

[77] Legendre L, Rueter S, Heinstein PF, Low PS (1993). Characterization of the oligogalacturonide-induced oxidative burst in cultured soybean (glycine max) cells. Plant Physiol.

[78] Boller T, Felix G (2009). A renaissance of elicitors: perception of microbe-associated molecular patterns and danger signals by pattern-recognition receptors. Annu Rev Plant Biol.

[79] Clough SJ, Bent AF (1998). Floral dip: a simplified method for Agrobacterium-mediated transformation of Arabidopsis thaliana. Plant J.

[80] Gómez-Gómez L, Felix G, Boller T (1999). A single locus determines sensitivity to bacterial flagellin in Arabidopsis thaliana. Plant J.

[81] Rumble SM, Lacroute P, Dalca AV, Fiume M, Sidow A, Brudno M (2009). SHRiMP: accurate mapping of short color-space reads. PLoS Comput Biol.

[82] Love MI, Huber W, Anders S. Moderated estimation of fold change and dispersion for RNA-Seq data with DESeq2. Genome Biol. 2014;15(12):550. 10.1186/s13059-014-0550-8 PMC4302049 25516281

[83] Vandesompele J, De Preter K, Pattyn F, Poppe B, Van Roy N, De Paepe A, et al. Accurate normalization of real-time quantitative RT-PCR data by geometric averaging of multiple internal control genes. Genome Biol. 2002;3(7):RESEARCH0034. 10.1186/gb-2002-3-7-research0034 PMC126239 12184808

[84] Beekman L, Tohver T, Dardari R, Léguillette R (2011). Evaluation of suitable reference genes for gene expression studies in bronchoalveolar lavage cells from horses with inflammatory airway disease. BMC Mol Biol.

[85] Ishiga Y, Ishiga T, Uppalapati SR, Mysore KS (2011). Arabidopsis seedling flood-inoculation technique: a rapid and reliable assay for studying plant-bacterial interactions. Plant Methods.

[86] Murashige T, Skoog F (1962). A revised medium for rapid growth and Bio assays with tobacco tissue cultures. Physiol Plant.

[87] Tateda C, Zhang Z, Shrestha J, Jelenska J, Chinchilla D, Greenberg JT. Salicylic Acid Regulates Arabidopsis Microbial Pattern Receptor Kinase Levels and Signaling. Plant Cell. 2014;26(10):4171-87. 10.1105/tpc.114.131938 PMC4247590 25315322

[88] Mootha VK, Lindgren CM, Eriksson K-F, Subramanian A, Sihag S, Lehar J (2003). PGC-1α-responsive genes involved in oxidative phosphorylation are coordinately downregulated in human diabetes. Nat Genet.

[89] Subramanian A, Tamayo P, Mootha VK, Mukherjee S, Ebert BL, Gillette MA (2005). Gene set enrichment analysis: A knowledge-based approach for interpreting genome-wide expression profiles. Proc Natl Acad Sci.

[90] Luo W, Brouwer C (2013). Pathview: an R/Bioconductor package for pathway-based data integration and visualization. Bioinformatics.

[91] Ortiz-Morea FA, Savatin DV, Dejonghe W, Kumar R, Luo Y, Adamowski M (2016). Danger-associated peptide signaling in *Arabidopsis* requires clathrin. Proc Natl Acad Sci.

[92] Merico D, Isserlin R, Stueker O, Emili A, Bader GD (2010). Enrichment Map: a network-based method for gene-Set enrichment visualization and interpretation. PLoS One.

