# Supplementary figures and images for: Short oligogalacturonides induce pathogen resistance-associated gene expression in Arabidopsis thaliana
Source: BMC Plant Biol. 2017 Jan 19;17:19. doi: 10.1186/s12870-016-0959-1 (PMC5248502; doi:10.1186/s12870-016-0959-1)

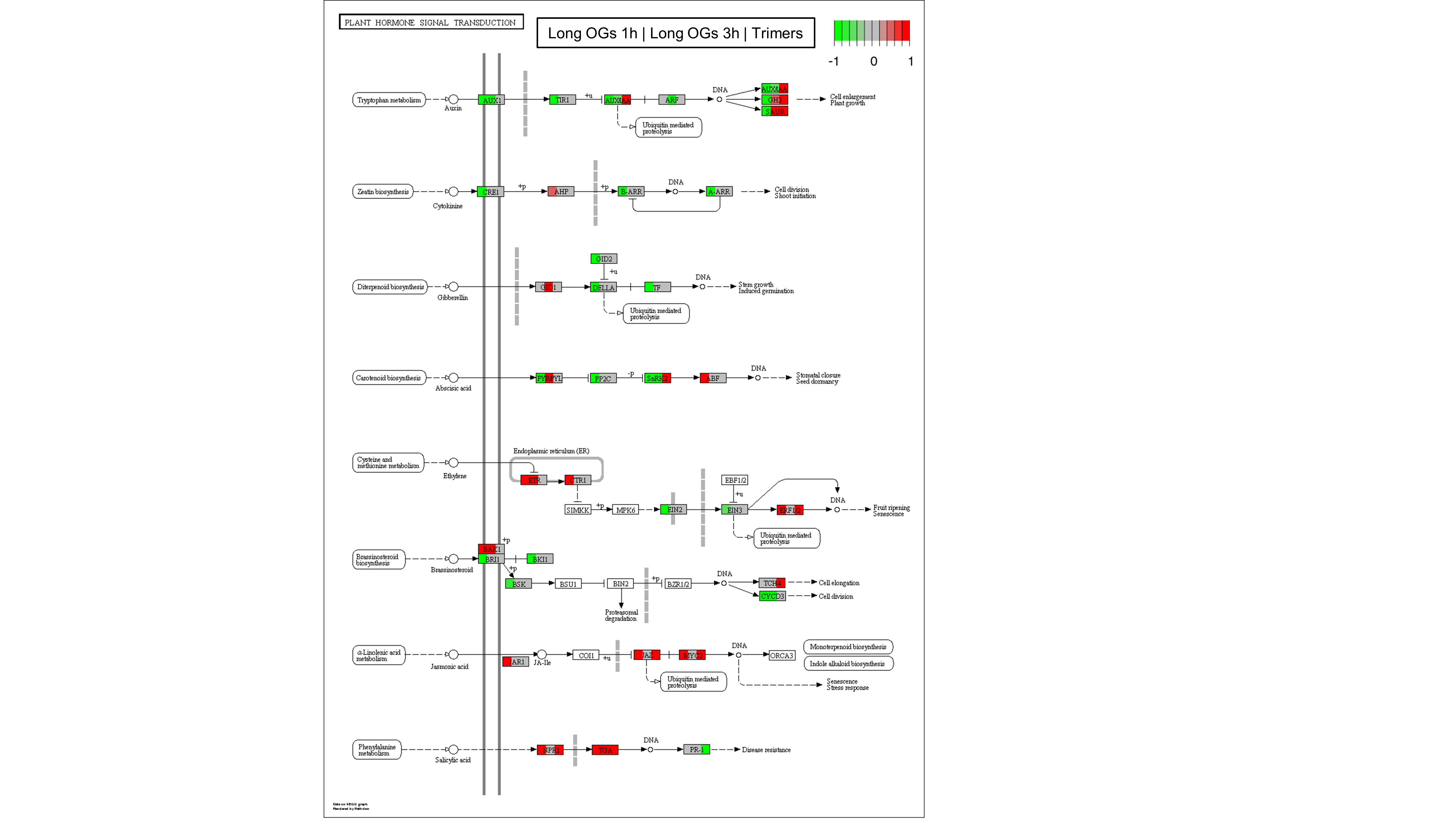

Supplement: Additional file 8: Figure S1. — Pathway analysis of plant hormone signal transduction pathways using RNA-sequencing data of plants treated by trimers compared to plants treated by a mock (Trimers). Also used is corresponding data from plants treated by long OGs at 1 h (long OGs 1 h) and 3 h (long OGs 3 h) (Denoux et al. 2008). (TIF 1568 kb) [file 12870_2016_959_MOESM8_ESM.tif]

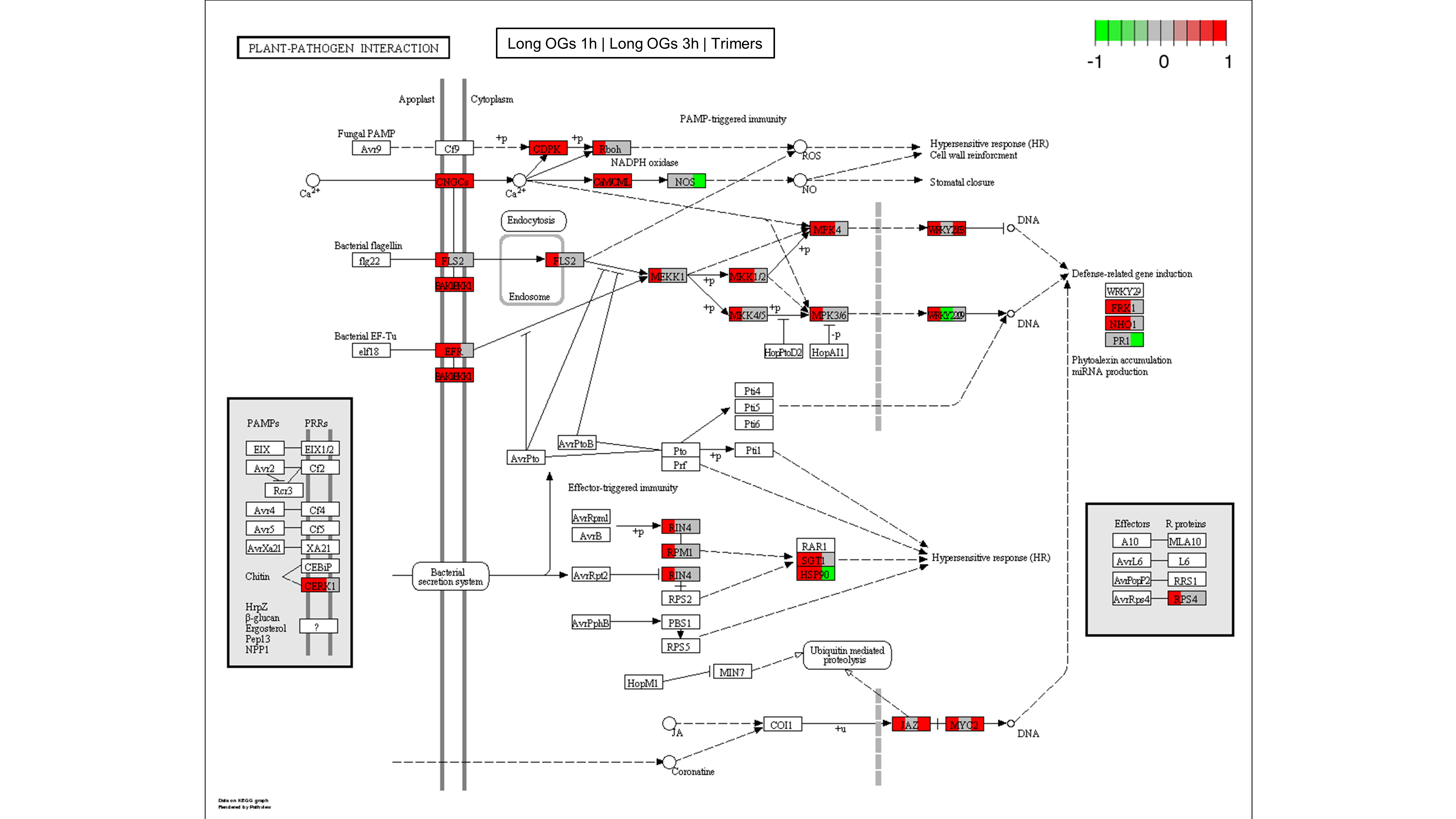

Supplement: Additional file 9: Figure S2. — Pathway analysis of plant-pathogen interaction pathways using RNA-sequencing data of plants treated by trimers compared to plants treated by a mock (Trimers). Also used is corresponding data from plants treated by long OGs at 1 h (long OGs 1 h) and 3 h (long OGs 3 h) (Denoux et al. 2008). (TIF 2399 kb) [file 12870_2016_959_MOESM9_ESM.tif]

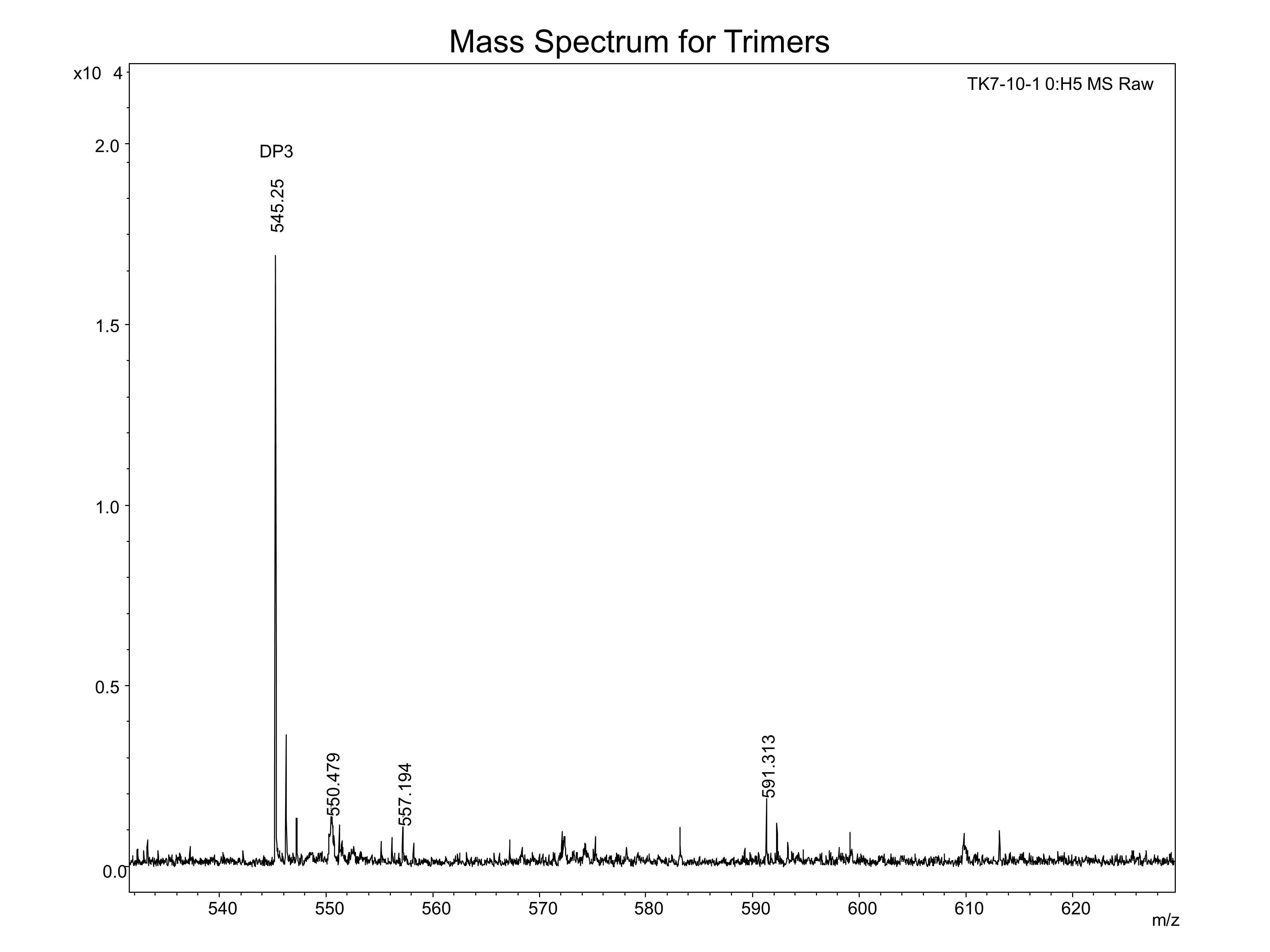

Supplement: Additional file 10: Figure S3. — Mass spectrum graph for the trimeric OGs used in this study (Trimers). (TIF 759 kb) [file 12870_2016_959_MOESM10_ESM.tif]

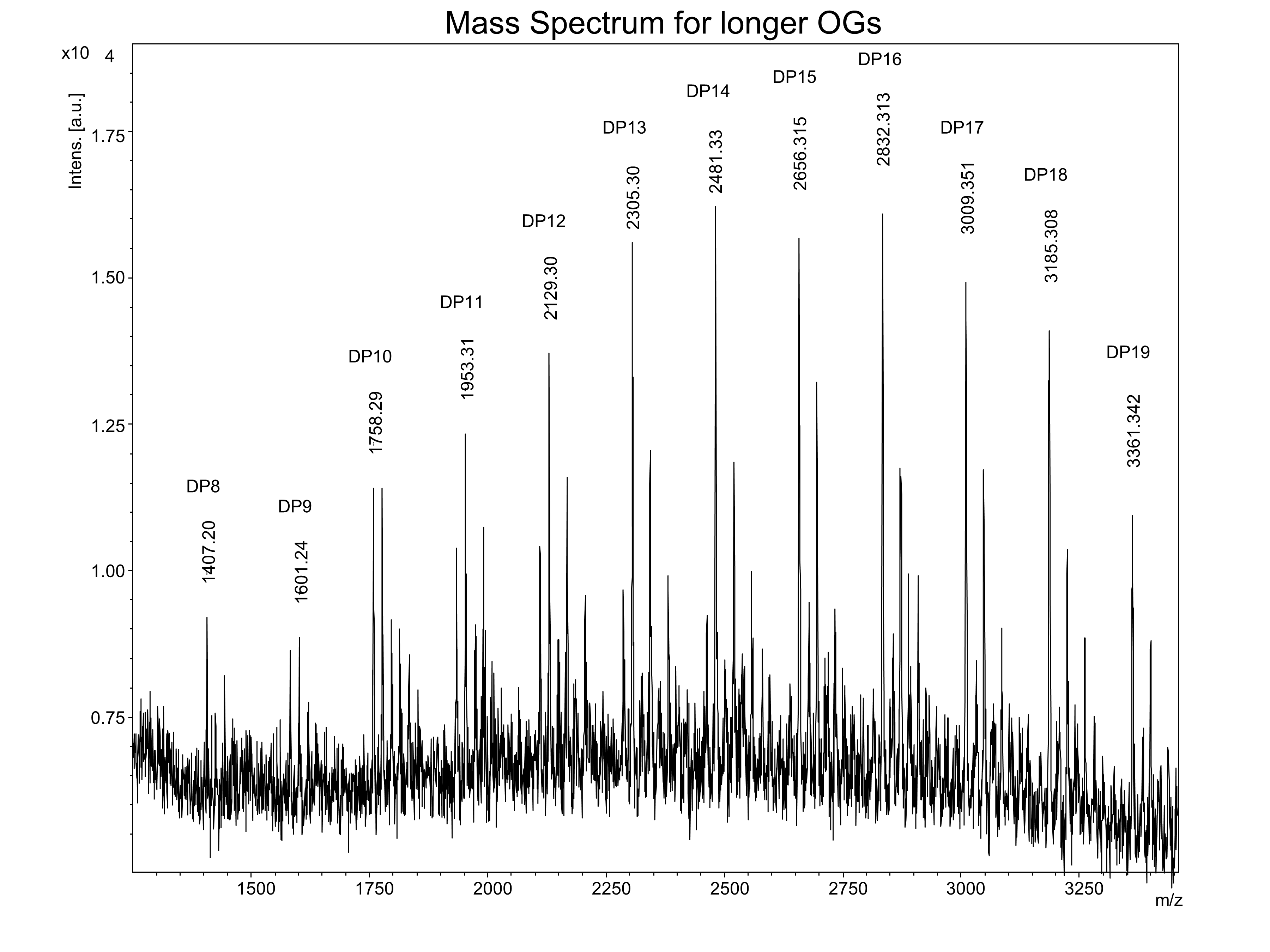

Supplement: Additional file 11: Figure S4. — Mass spectrum graph for the long OGs with a degree of polymerization >8 used in this study (long-OG mix). (TIF 1425 kb) [file 12870_2016_959_MOESM11_ESM.tif]

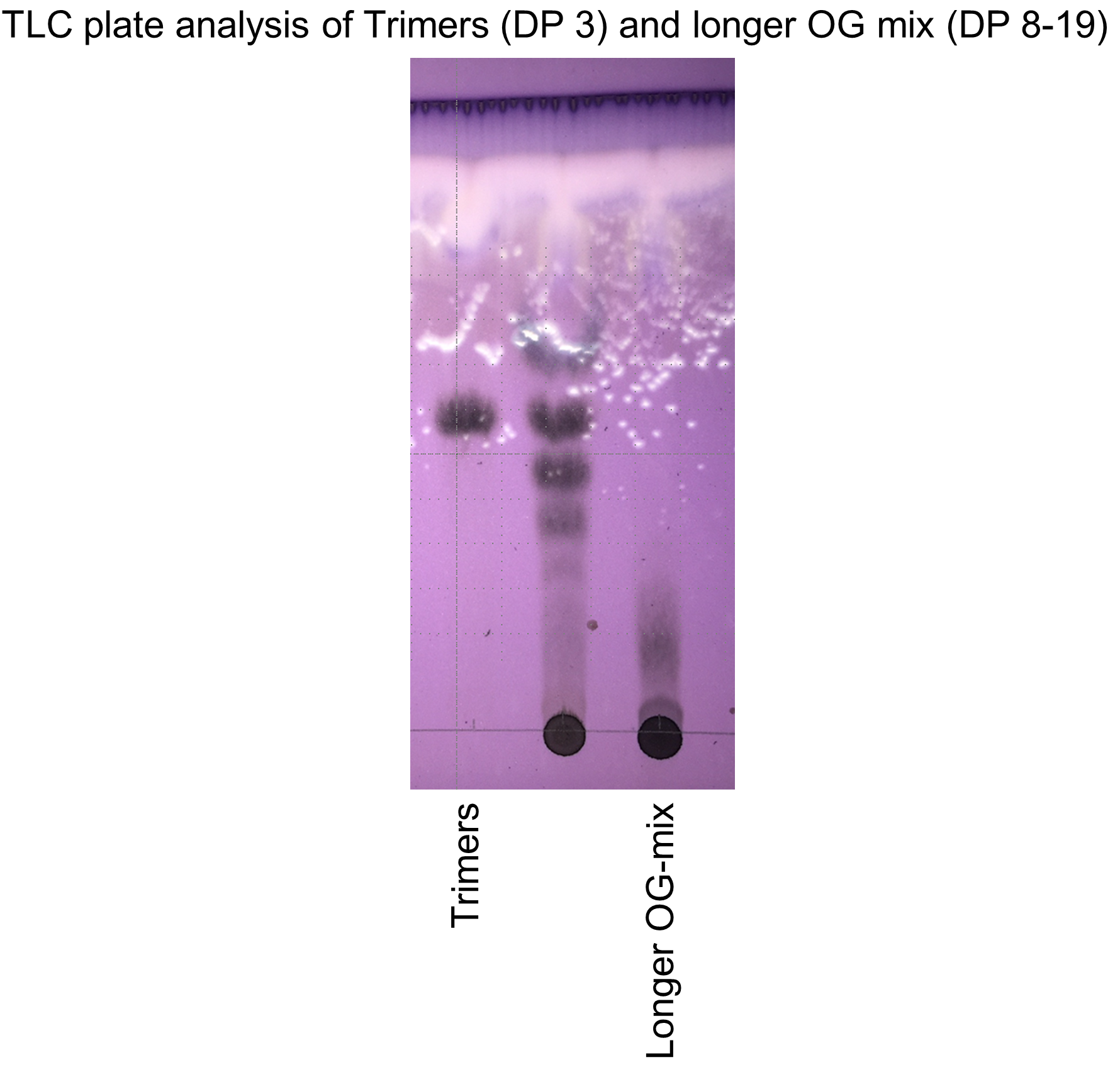

Supplement: Additional file 12: Figure S5. — Results of TLC plate analysis of Trimers and long-OG mix (longer OG mix). (TIF 973 kb) [file 12870_2016_959_MOESM12_ESM.tif]
